# Oxygen tension modulates the mitochondrial genetic bottleneck and influences the segregation of a heteroplasmic mtDNA variant in vitro

**DOI:** 10.1038/s42003-021-02069-2

**Published:** 2021-05-14

**Authors:** Mikael G. Pezet, Aurora Gomez-Duran, Florian Klimm, Juvid Aryaman, Stephen Burr, Wei Wei, Mitinori Saitou, Julien Prudent, Patrick F. Chinnery

**Affiliations:** 1 Department of Clinical Neurosciences, School of Clinical Medicine, University of Cambridge, Cambridge Biomedical Campus, Cambridge, CB2 0QQ UK; 2 Medical Research Council Mitochondrial Biology Unit, University of Cambridge, Cambridge Biomedical Campus, Cambridge, CB2 0XY UK; 3 Department of Mathematics, Imperial College London, London, SW7 2AZ UK; 4 Department of Anatomy and Cell Biology, Graduate School of Medicine, Kyoto University, Kyoto, 606-8501 Japan; 5 JST, ERATO, Kyoto University, Kyoto, 606-8501 Japan; 6 Present Address: Department of Medicine, Columbia University Medical Center, New York, NY USA

**Keywords:** Cell biology, Metabolic disorders

## Abstract

Most humans carry a mixed population of mitochondrial DNA (mtDNA heteroplasmy) affecting ~1–2% of molecules, but rapid percentage shifts occur over one generation leading to severe mitochondrial diseases. A decrease in the amount of mtDNA within the developing female germ line appears to play a role, but other sub-cellular mechanisms have been implicated. Establishing an in vitro model of early mammalian germ cell development from embryonic stem cells, here we show that the reduction of mtDNA content is modulated by oxygen and reaches a nadir immediately before germ cell specification. The observed genetic bottleneck was accompanied by a decrease in mtDNA replicating foci and the segregation of heteroplasmy, which were both abolished at higher oxygen levels. Thus, differences in oxygen tension occurring during early development likely modulate the amount of mtDNA, facilitating mtDNA segregation and contributing to tissue-specific mutation loads.

## Abstract (web summary)

Using an in vitro culture system, Pezet et al. studied the influence of oxygen on the mitochondrial DNA (mtDNA) in primordial germ cell-like cells (PGCLCs) in vitro. Low oxygen levels resembling in vivo reduced the cell mtDNA content causing a genetic bottleneck and the segregation of different mtDNA genotypes.

## Introduction

Mammalian cells contain many copies of the circular mitochondrial DNA (mtDNA), which codes for 13 proteins required for oxidative metabolism and the synthesis of adenosine triphosphate (ATP)^1^. mtDNA is almost exclusively maternally inherited, and until recently, it was assumed that in most humans all of mtDNA was identical (homoplasmic wild-type; WT). However, single-cell and massively parallel bulk mtDNA sequencing has shown the converse—that most if not all humans have a mixed population of mtDNA molecules (heteroplasmy)^2^. Usually these mutations only affect the minority of mtDNA molecules (~1–2%), falling well below the critical threshold (50–85%) typically required to cause a cellular biochemical defect^1,2^. However, rapid changes in heteroplasmy are seen during maternal transmission, explaining why an unaffected woman carrying a pathogenic mutation can have a child with a severe mtDNA disease, known to affect ~1 in 8500 of the population^3^. In patients with mtDNA diseases, affected cells and organs have higher levels of heteroplasmy, but it is not clear how the tissue-specific segregation arises during development^2^. Heteroplasmic mtDNA mutations have also been implicated in the pathogenesis of common late-onset disorders including neurodegenerative diseases^4^, and even the ageing process itself^5^, so the mechanisms of heteroplasmy segregation have far-reaching implications.

Rapid shifts in heteroplasmy were first seen in Holstein cows leading to the mitochondrial bottleneck hypothesis^6^, where only a small proportion of the maternal mitochondria contribute to the next generation. The bottleneck is thought to cause a sampling effect resulting in different proportions of mutant and WT molecules in each offspring. Heteroplasmy measurements across several species implicate a mitochondrial bottleneck during female germ cell development^7,8^, but the precise mechanism is a source of debate. Several studies have measured an evolutionary conserved physical reduction in cellular mtDNA content within the developing germ line^8–12^. Statistical genetic theory and in silico modelling show the measured reduction in mtDNA content is sufficient to cause different heteroplasmy levels in siblings^13^, but there is no direct experimental evidence showing that the reduction in mtDNA levels is essential for mtDNA segregation. Moreover, the variation in heteroplasmy levels (heteroplasmy variance) predicted by the models only accounts for ~70% of the observed variance seen in offspring^10^, implicating mechanisms other than the simple reduction of mtDNA copy number.

Although the total amount of mtDNA within the embryo remains constant during pre-implantation development^10,11,14^, there is ongoing mtDNA turnover in pre-implantation embryos^15,16^. This means that the measured reduction of mtDNA within individual cells could be due to several mechanisms, including a reduction of the replication rate of mtDNA and an increase in the destruction of mtDNA by autophagy^16^. Resolving these issues at a cellular level will be challenging in living organisms. For this reason, we developed an in vitro model of the critical time point during early germ cell development, allowing us to study mitochondria and mtDNA at the cellular and sub-cellular level during and immediately before germ cell specification.

## Results

### Low oxygen tension generates a genetic bottleneck before PGCLC specification and modulates heteroplasmy segregation

Embryonic stem cells (ESCs) were isolated from mice carrying two transgenic reporter genes, *Blimp1-mVenus (BV)* and *Stella-ECFP* (*SC*), allowing the identification and isolation of primordial germ cell-like cells (PGCLCs) throughout their in vitro development^17,18^. Using ultra-high-depth mtDNA sequencing (mean coverage 2869-fold, SD: 909) of *BVSC* ESC, we detected heteroplasmic mtDNA variants (Supplementary Data 3), as seen in human stem cell lines^19^. We subcloned a *BVSC* ESC line (Fig. S1a) and identified an ESC clone that did not contain detectable heteroplasmic mtDNA variants (homoplasmic WT), and a second ESC clone harbouring a highly conserved heteroplasmic variant in *mt-Nd1* (m.3062T > A/*mt-Nd1*, ND1:p.F104V) (Fig. S1b and Supplementary Data 3) with a mean heteroplasmy of 29.6% stable over five passages before differentiation (thereafter called ND1) (Fig. S1c). The ND1 cell line had a 17% reduction in oxygen consumption compared to the WT (Fig. S1d), with no difference in glycolysis activation (Fig. S1e), consistent with mild defect of mitochondrial respiration.

**Fig. 1:**
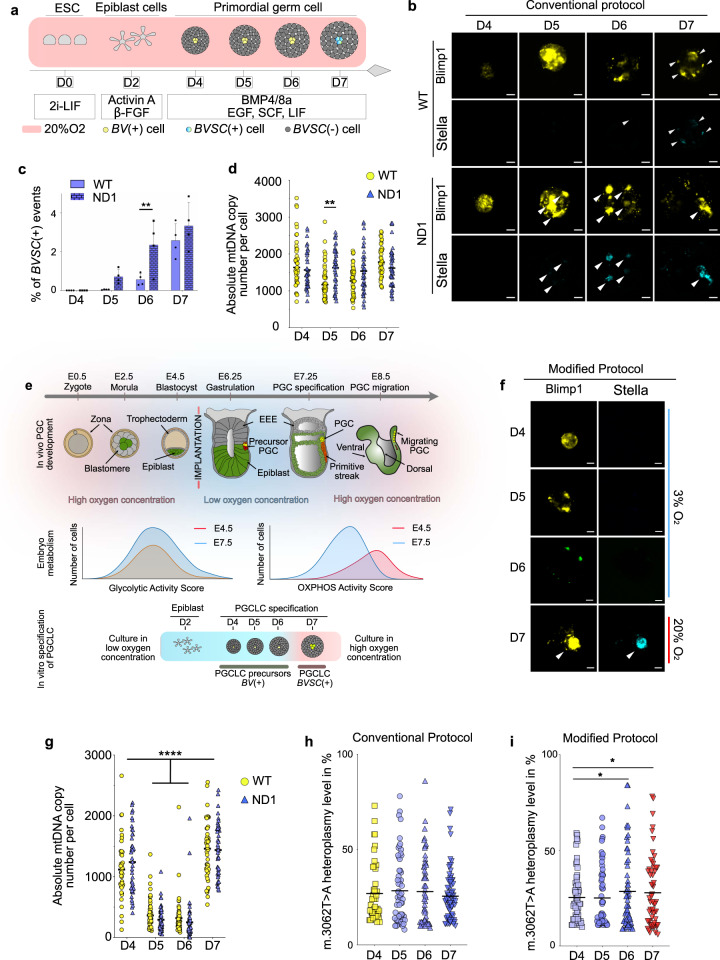
**Low oxygen tension generates a genetic bottleneck before PGCLC specification and modulates the heteroplasmy segregation**. **a** Scheme highlighting the conventional protocol for PGCLC differentiation. **b** Differentiation monitoring at 20% oxygen. Representative microscopy confocal imaging. White arrows show PGCLCs. Scale bars = 100 µm. **c** Percentages of the *BVSC*(+) events acquired by FACS (mean ± SEM, *n* = 4 independent differentiation, ^**^p < 0.01, two-way ANOVA with multiple comparisons using Sidak test). **d** Number of mtDNA molecules per single cell. Horizontal lines represent mean, ^**^p < 0.01, two-way ANOVA with multiple comparisons using Sidak test (for WT cells, D4: 41 cells; D5: 45 cells; D6: 43 cells; D7: 40; for ND1 cells, D4: 37 cells; D5: 40 cells; D6: 39 cells; D7: 43 cells from 3 independent differentiations). **e** Cartoon representing primordial germ cell (PGC) specification in vivo (top panel) illustrating the local oxygen levels during pre- and post-implantation development. The intermediate panel summarises the metabolism of embryos at E4.5 and E7.5 based on the transcriptomic analysis performed and shown in Fig. S2a. The bottom panel shows the modified in vitro protocol for specification of PGCLCs, modelling the increased oxygen requirement in vivo. In vitro epiblast-like cells (EpiLCs), epiblast-like cells in vivo, begin differentiation in a low oxygen concentration. From D6 to D7, the oxygen concentration is increased, which is essential for *Stella* expression in PGCLCs corresponding to PGCs at E7.25. Blue = low oxygen, red = high oxygen. **f** Monitoring of the differentiation of ND1 cells performed at various concentrations of oxygen. Representative microscopy confocal imaging. White arrows show PGCLCs. Scale bars = 100 µm. **g** Number of mtDNA molecules per single WT and ND1 cells using the modified protocol of differentiation. Horizontal lines represent mean, ^****^p < 0.0001 two-way ANOVA with multiple comparisons using Sidak test (WT, D4: 40 cells; D5: 57 cells; D6: 56 cells; D7: 45; ND1, D4: 44 cells; D5: 61 cells; D6: 63 cells; D7: 44 cells from 3 independent differentiations). **h** Heteroplasmy measurements of m.3062T > A:p.mt-ND1 per cell in *BV* (yellow) and in *BVSC* (blue range)-positive cells during differentiation at 20% oxygen (D4: 70 cells, *n* = 4, variance: 0.024; D5: 63 cells, *n* = 4, variance: 0.031; D6: 59 cells, *n* = 4, variance: 0.032; D7: 76 cells, *n* = 4, variance: 0.017). **i** Heteroplasmy measurements of m.3062T > A:p.mt-ND1 per cell in *BV*(+) (blue range at D4, D5 and D6 at 3% oxygen concentration) and in *BVSC*(+) (red, at D7 at 20% oxygen concentration) cells (D4: 46 cells, *n* = 3, variance: 0.020; D5: 60 cells, *n* = 4, variance: 0.025; D6: 58 cells, *n* = 4, variance: 0.041; D7: 45 cells, *n* = 3, variance: 0.036). The horizontal lines represent mean, ^*^p > 0.1 with a one-sided bootstrap confidence interval test (50k iterations) for whether the variance was greater at a later time point relative to D4, with Benjamini-Hochberg correction with false-discovery rate = 0.1.

The conventional protocol for in vitro PGCLC differentiation is performed at 20% oxygen and requires the specification of ESC to epiblast-like cells (EpiLCs, for 2 days, D2) (Figs. 1a and S1f) followed by the expression of *BV* (from D3 onwards) and *SC* (Fig. 1a), recapitulating the in vivo gene expression profile of primordial germ cells (PGCs)^20^. In order to investigate the segregation of the heteroplasmic ND1 variant during the early stages of PGC development, PGCLCs were generated using the two isogenic ESC subclones (WT and ND1, Fig. 1b)^21^ that sequentially expressed the *BV* followed by the *SC* transgenic reporter genes (Fig. 1c and S1g, h). Measuring the number of *BVSC*(+) cells isolated at the same time point showed a reduced rate of proliferation for the differentiating ND1 line, consistent with the observed oxygen consumption defect (Fig. S1i). The mtDNA content was measured in single *BV*(+) and *BVSC*(+) cells from 3 independent experiments (Fig. 1d and Supplementary Data 1). ND1 cells showed a constant mtDNA content while the WT cells displayed a slight decrease at D5 of differentiation in comparison to their counterpart (Fig. 1d and Supplementary Data 1). The mtDNA content on both cell lines was ~5-fold greater than the ~200 copies per cell measured in early mouse PGCs in vivo^9–11^, indicating that germ cell specification does not, on its own, lead to a mtDNA genetic bottleneck.

In utero, the oxygen concentration progressively goes down as the embryo reaches the uterus (Fig. 1e)^22,23^, in line with in vitro studies showing that a low oxygen concentration leads to a higher efficiency of early embryo development. During this period of pre-implantation development, the mtDNA content of the whole embryo remains constant, while the mtDNA copy number per cell drops^10,11,24^. Vascularisation of the embryo occurs post-implantation, between ~E6.5 and E9.5^25,26^, which also corresponds to the time of PGCs specification. Consequently, the oxygen concentration within the embryo progressively increases^22^ in order to support its development. We speculated that the reduction of oxygen concentration during the early stages of the embryo development was required for the decrease of the mtDNA content. To support this, we took advantage of available single-cell (sc) RNA-seq data collected from whole mouse embryos at E4.5 and E7.5^27^. Our re-analysis showed that at E4.5, blastocysts expressed significantly more OXPHOS-related genes than later stage E7.5 embryos (*p*-value = 10^−1^ ^24^; Figs. 1e and S2a), consistent with a lower oxygen concentration at E7.5 coinciding with the mtDNA bottleneck in vivo. To model the variation of oxygen levels during the early developmental stages in vivo (Fig. 1e), we adapted the differentiation protocol so that the first four days of the PGCLC specification were at low (3%) oxygen level, followed by high (20%) oxygen level for the last 24 h.

As a first step, we maintained ESCs at 3% oxygen. This led to a stable decrease in mtDNA content (Fig. S2a–d) without decreasing the mitochondrial mass (Fig. S2e) as seen previously^28^. To determine whether low oxygen levels could lead to a genetic bottleneck during differentiation, we used the modified protocol (Figs. 1e, f and S3a) to isolate *BV*(+) (from D4 to D6) and *BVSC*(+) (at D7) cells by fluorescence-activated cell sorting (FACS) from both the WT and ND1 lines from 4 independent experiments (Fig. S3b–d). Analysis of the mtDNA content in single cells measured from WT *BV*(+) cells showed an ~4-fold decrease in the mean mtDNA content from 1119 (SD: ±495 copies) at D4 to 319 (SD: ±300 copies) at D6 (Fig. 1g and Supplementary Data 1). We saw a similar pattern for the ND1 cells (4-fold, 1240 copies, SD: ±513 copies at D4; 249 copies, SD: ±294 copies at D6) (Fig. 1g and Supplementary Data 1). At D7 of differentiation, the mtDNA copy number increased to 1434 (SD: ±495 copies) and 1462 (SD: ±513 copies) in WT and ND1 *BVSC*(+), respectively (Fig. 1g and Supplementary Data 1). *SC* expression and the increase in mtDNA content were prevented if the cells were maintained at low oxygen concentrations beyond D6 (Fig. S3e–g). Importantly, the culture conditions at low oxygen concentration did not affect cell viability (Fig. S3h), nor the subsequent expression of *BVSC*, which was similar to cells differentiated in 20% oxygen.

Given our previous findings, next we determined whether the reduction of mtDNA copy number generated by low oxygen level influenced the segregation of mtDNA heteroplasmy. We studied single-cell measurements taken throughout the differentiation process of the heteroplasmic ND1 line. In 20% oxygen, when the cells maintained their mtDNA content (Fig. 1h and Supplementary Data 1), the heteroplasmic mt-ND1 variant did not segregate. However, in 3% oxygen, the observed reduction in mtDNA content in *BVSC*(+) cells was accompanied by an increase in the heteroplasmy variance (Fig. 1i and Supplementary Data 1). Taken together, our data suggest that oxygen concentration modulates the heteroplasmy segregation.

### The genetic bottleneck is accompanied by a reduction in the number of mtDNA-replicating foci, but no activation of autophagy

**Fig. 2:**
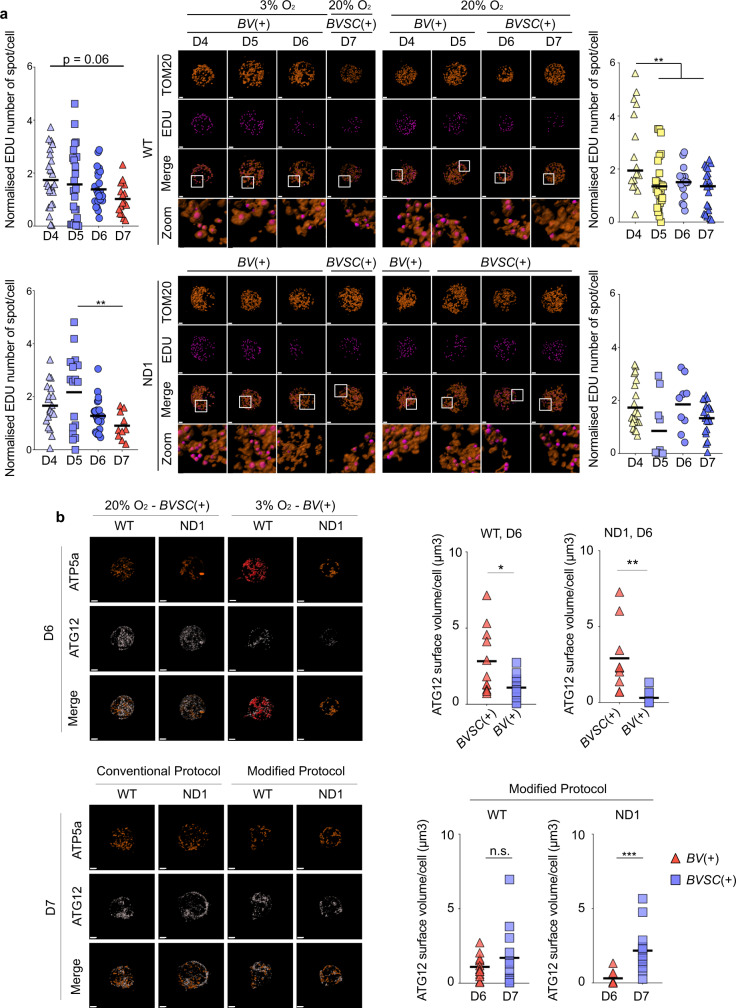
**The genetic bottleneck is associated with a reduction of mtDNA replication, but no autophagy activation**. **a** Representative microscopy confocal staining images of TOM20 surface (red, upper) and pulse-labelled with EDU spot (purple, lower) surfaces generated by IMARIS software based on fluorescent intensity signal of *BV*(+) and *BVSC*(+) cells during PGCLC differentiation. Scale bars = 2 µm. Original images showing the fluorescent intensity are shown in Fig. S4c. Respective quantification of the relative number of EDU spots per cell are shown (colour code of the left panel reflects the concentration of oxygen; colour code of the right panel reflects the expression of *BV* (yellow) and *BVSC*(blue) cells). Horizontal bars represent the mean, ^*^p < 0.05, ^**^p < 0.01, ^***^p < 0.001 and ^****^p < 0.0001, one-way ANOVA with multiple comparisons using Tukey test relative to D7 (WT 3% O_2_, D4: 25 cells, *n* = 3; D5: 24 cells, *n* = 3; D6: 26 cells, *n* = 3; D7: 16 cells, *n* = 2; ND1 3% O_2_, D4: 19 cells, *n* = 3; D5: 17 cells, *n* = 3; D6: 24 cells, *n* = 3; D7: 13 cells, *n* = 2; WT 20% O_2_, D4: 16 cells, *n* = 3; D5: 25 cells, *n* = 3; D6: 15 cells, *n* = 3; D7: 20 cells, *n* = 3; ND1 20% O_2_, D4: 23 cells, *n* = 3; D5: 10 cells, *n* = 2; D6: 9 cells, *n* = 2; D7: 20 cells, *n* = 3). **b** Representative microscopy confocal staining images of ATP5a surface (red) and ATG12 surfaces (white) generated by IMARIS software based on fluorescent intensity signal of *BV*(+) and *BVSC*(+) cells at D6 and D7 of differentiation of WT and ND1 using either the conventional or the modified protocol. Scale bars = 2 µm. Original images showing the fluorescent intensity are available in Fig. S5a. ATG12 surface volume per WT and ND1 cells quantifications at 20% O_2_ (red, *BVSC*(+)) and 3% O_2_ (blue, *BV*(+)) at D6 of differentiation shown on the top right panels. The horizontal lines represent the mean, ^*^p < 0.05, ^**^p < 0.01 Student’s *t*-test (WT-*BVSC*(+): 11 cells, *n* = 2; WT-*BV*(+): 14 cells, *n* = 2; ND1-*BVSC*(+): 9 cells, *n* = 2; ND1-*BV*(+): 9 cells, *n* = 2). ATG12 surface volume comparison BV(+) cells (red, 3% oxygen, D6) and *BVSC*(+) cells (blue, 20% oxygen for 24 h, D7) cells shown on the bottom-right panel (‘Modified Protocol’). The horizontal lines represent the mean, ^***^p < 0.001, Student’s *t*-test (WT: D6: 14 cells, *n* = 3; D7: 12 cells, *n* = 3; ND1: D6: 9 cells, *n* = 2; D7: 18 cells, *n* = 3).

The decrease of mtDNA content per cell during the early stages of embryogenesis is believed to be due to the sub-compartmentalisation of a fixed number of mtDNA molecules with each cell division^10,11^. However, given evidence of ongoing mtDNA turnover^15^, other mechanisms could also play a part. First, we measured the cell proliferation rate during PGCLC differentiation which did not correlate directly with cell-mtDNA levels (Fig. S4a, b), pointing towards other mechanisms. Next, we studied mtDNA replication using the thymidine analogue 5-ethynyl-2′-deoxyuridine (EDU)^29^ which co-localised with the mitochondrial outer-membrane marker TOM20 at the single-cell level (Figs. 2a and S4c, Supplemental Video 1 and Supplementary Data 1). Each cell line was pulsed with EDU for 30 min, enabling a measurement of the number of active replicating foci, which reflects the mtDNA replication rate^30^. At 3% oxygen_,_ the number of replicating foci per cell showed a progressive trend to decrease in both WT and ND1 *BV*(+) cells (Figs. 2a and S4c, and Supplementary Data 1), in keeping with the ~4-fold decrease of mtDNA content. The replication of the mtDNA in the WT cells at 20% O_2_ was also reduced from D4 to D5, but less so, corresponding to the slight reduction of mtDNA observed at D5 (Figs. 1d, 2a and S4c, and Supplementary Data 1). By contrast, the number of replicating foci in the ND1 *BVSC*(+) cells remained unchanged at 20% oxygen, consistent with the constant mtDNA copy number observed during the differentiation (Figs. 2a and S4c, and Supplementary Data 1). Taken together, this showed that the reduction of mtDNA copy number in cells correlates with a decrease of mtDNA replication rate in keeping with this playing a role in the bottleneck mechanism. However, the lack of a complete correspondence between replicating foci and mtDNA levels, particularly on D7, indicates that other mechanisms must also come in to play.

Intensive fragmentation of the mitochondrial network would allow the degradation of mtDNA molecules through the autophagy of individual mitochondria^16^, and thus contribute to the bottleneck mechanism. In keeping with this, we observed fragmentation of the mitochondrial network in *BV*(+) cells at low oxygen concentrations, with a significant reduction of the mean length of mitochondria (Fig. S4c). To determine whether autophagy was playing a role in the mtDNA bottleneck, we studied ATG12 which is recruited to the pre-autophagosomal structure^31,32^. In both the WT and ND1 cell lines, at D6, *BV*(+) cells containing a low level of mtDNA also expressed low levels of ATG12 protein (Figs. 2b and S5a, and Supplementary Data 1). The increased mtDNA content at D6 to D7 (Fig. 1g) when cells were differentiated using the modified protocol occurred along with an increase of ATG12 level (Figs. 2b and Fig. S5a, and Supplementary Data 1). Together, these findings make it unlikely that ATG12-mediated autophagy is involved in the regulation of mtDNA content during PGC differentiation.

### Unequal partitioning of mitochondria in *BVSC*(−) cells

In addition to the well-established germ line genetic bottleneck, several studies have shown a reduction of mtDNA content in somatic tissues during early development^8,9,12^. Potentially, this could contribute to the segregation of mtDNA heteroplasmy in developing tissues and organs, leading to high mutation loads in vulnerable tissues, which cause disease^33^. To explore this, we studied *BVSC*(−) single cells isolated from WT and ND1 cell lines throughout their specification process (Fig. S6a). It is likely that this cell population is heterogeneous, and although we did not characterise them in detail, a proportion are likely to develop into committed somatic tissues^20^. At 20% oxygen, in contrast to the PGCLCs, we saw a 1.5- and 2.5-fold-change decrease in mtDNA levels in the WT and ND1 cell lines, respectively (WT D4: mean of 1027 copies, SD: ±535 copies; WT D6: mean of 690 copies, SD: ±306 copies; ND1 D4: mean of 1559 copies, SD: ±606 copies; ND1 D6: mean of 606 copies, SD: ±223 copies corresponding to a >1.5-fold-change decrease in WT *BVSC*(−) and >2.5-fold-change decrease in ND1 *BVSC*(−), respectively; Fig. S6b, c). However, at low oxygen tensions (3%), we observed a >4-fold decrease of mtDNA content in *BVSC*(−), similar to the adjacent PGCLCs at D6 (WT D4: mean of 1003 copies, SD: ±472 copies; WT D6: mean of 212 copies, SD: ±160 copies; ND1 D4: mean of 1207 copies, SD: ±494 copies; ND1 D6: mean of 133 copies, SD: ±106 copies; Fig. S6d, e). These findings resemble observations in vivo where somatic cell genetic bottlenecks have also been shown in mice, zebrafish and humans^8,9,12^, but show that the size or ‘severity’ of the genetic bottleneck is modulated in part by the oxygen concentration in *BVSC*(−) cells (Fig. S6f).

**Fig. 3:**
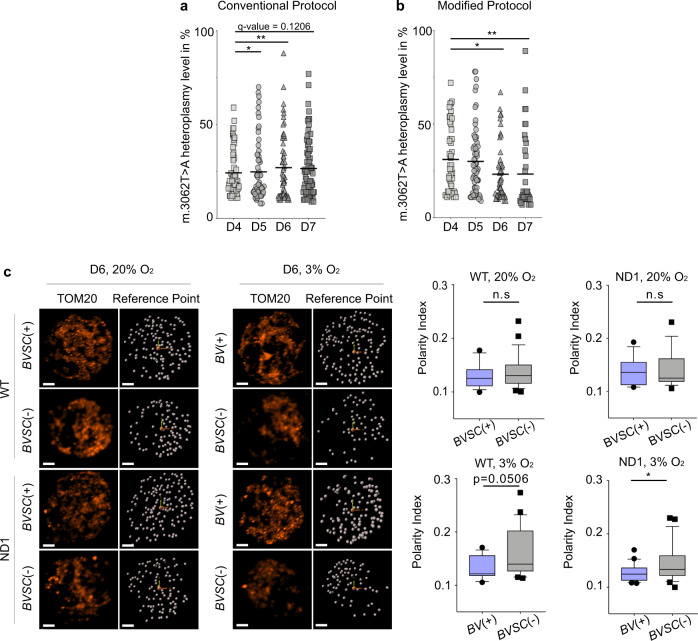
**Tissue-specific mechanism of segregation: unequal partitioning of mitochondria in*BVSC*(−)**. **a** Heteroplasmy measurements of m.3062 T > A:p.mt-nd1 per cell in *BVSC*(−) cells during differentiation at 20% oxygen (D4: 55 cells, *n* = 4, variance: 0.015; D5: 64 cells, *n* = 4, variance: 0.027; D6: 58 cells, *n* = 4, variance: 0.032; D7: 75 cells, *n* = 4, variance: 0.024). **b** Heteroplasmy measurements of m.3062T > A:p.mt-nd1 per cell in *BVSC*(−) cells during differentiation at 3% oxygen (D4: 47 cells, *n* = 3; D5: 61 cells, *n* = 4; D6: 51 cells, *n* = 4; D7: 39 cells, *n* = 3). The horizontal lines represent mean, ^*^p > 0.1, ^**^p > 0.05 with a one-sided bootstrap confidence interval test (50k iterations) for whether the variance was greater at a later time point relative to D4, with Benjamini-Hochberg correction with false-discovery rate = 0.1. **c** Representative mitochondrial distribution (TOM20, red) in *BVSC*(−), *BV*(+) and *BVSC*(+) cells at D6 in both WT and ND1 cells when cultured at either 20% or 3% oxygen. Mitochondrial spots on the surface (white dots) were counted in the 3D space according to a reference point. Respective quantifications are shown on the right panels. The data represent a box plot, ^*^p < 0.05 Student’s *t*-test, (WT *BVSC*(+) 20% O_2_: 16 cells, *n* = 3; WT *BVSC*(−) 20% O_2_: 16 cells, *n* = 2; ND1 *BVSC*(+) 20% O_2_: 18 cells, *n* = 3; ND1 *BVSC*(−) 20% O_2_: 22 cells, *n* = 3; WT *BV*(+) 3% O_2_: 23 cells, *n* = 3; WT *BVSC*(−) 3% O_2_: 21 cells, *n* = 3; ND1 *BV*(+) 3% O_2_: 16 cells, *n* = 3; ND1 *BVSC*(−) 3% O_2_: 22 cells, *n* = 3).

Next, we studied the consequences of oxygen concentration on the segregation of *ND1* heteroplasmy in the *BVSC*(−) cells. As expected, at 20% oxygen levels, the presence of a genetic bottleneck was linked to heteroplasmy segregation (Fig. 3a and Supplementary Data 1). However, at 3% oxygen we also saw a decrease in the median heteroplasmy level from D4 (median = 26) to D7 (median = 12, *p* = 0.0065) consistent with negative selection acting against the m.3062T > A/*mt-Nd1* variant in somatic cells (Fig. 3b and Supplementary Data 1). Hence, the oxygen tension altered the outcome of the heteroplasmy segregation in *BVSC*(−) cells. As expected, the mtDNA replication rate was decreased in both 20% and 3% of oxygen in keeping with a reduced mtDNA content (Fig. S6g). Moreover, ATG12 levels in *BVSC*(−) cells were found to be lower than in *BV*(+) and *BVSC*(+) emphasising an autophagy-independent mechanism responsible for mtDNA genetic bottleneck (Fig. S5a–c). It also suggested that autophagy was not involved during the negative selection of the heteroplasmic mt-ND1 variant observed at 3% O_2_ despite displaying fragmented mitochondria (Fig. S7).

The unequal partitioning of mitochondria has been proposed to contribute to heteroplasmy segregation^11^. We therefore investigated the distribution of mitochondria during differentiation in *BV*(+), *BVSC*(+) and *BVSC*(−) cells using the IMARIS referential frame reduction function to study the cells in three-dimensions. The mitochondria remained evenly distributed throughout the cytoplasm in both the WT and ND1 cells at 20% oxygen level (Fig. 3c). However, at 3% oxygen, the WT and ND1 *BVSC*(−) cells had more polarised mitochondria compared to the *BV*(+) cells (Fig. 3c). Thus, in our in vitro model, the asymmetric compartmentalisation of mitochondria could also contribute to the segregation of mtDNA heteroplasmy in *BVSC*(−) cells at low oxygen concentrations. Although unlikely to play an important role during early PGCLC development, we cannot exclude asymmetric compartmentalisation being at stake in germ line heteroplasmy segregation at later stages.

### Oxygen tension modulates the expression of genes involved in the replication of mtDNA, but not autophagy

To further investigate the impact of oxygen tension upon mtDNA replication, we performed sc-RNA-seq during in vitro differentiation of WT cells from D4 to D7 in 3 independent experiments at 20% and 3% of oxygen (Fig. 1a, e). In total, we analysed the transcriptome from 740 single cells.

**Fig. 4:**
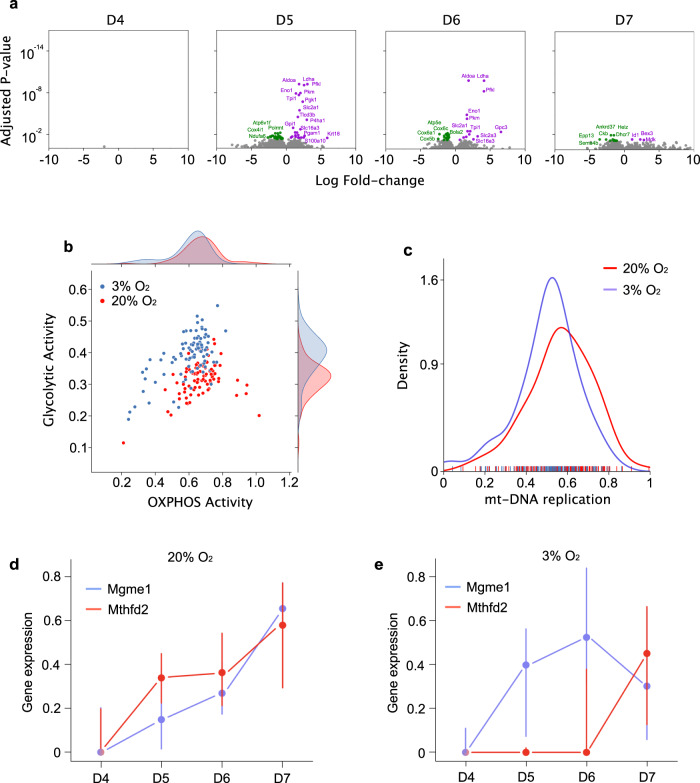
**Single-cell RNA sequencing in*BVSC(*+*/−)*cells during WT cell differentiation in high and low oxygen concentrations**. **a** Volcano plots showing the differentially expressed genes for each day between positive cells differentiating with the conventional (20% oxygen) and the modified (3% oxygen) protocol. Coloured dots represent significantly up-regulated (purple) and down-regulated (green) genes with multiple-testing corrected *p*-value < 0.05, as identified by Wilcoxon rank-sum tests (20% O2: D4: 47 cells, D5: 40 cells, D6: 45 cells, D7: 48 cells, *n* = 3; 3% O2: D4: 40 cells, D5: 48 cells, D6: 48 cells, D7: 48 cells, *n* = 3). **b** Scatter plots with marginal distributions showing the metabolism-related gene expressions in PGCLC at D5 and D6 (data combined together) when differentiated at low (blue) and high (red) oxygen concentration. Each dot represents a single cell. Its (*x*,*y*) coordinates are calculated by the number of genes expressed related to glycolytic (*y* axis) and OXPHOS (*x* axis) metabolism. Data consider the expression of 85 glycolytic genes and 96 OXPHOS genes (Supplementary Data 2). OXPHOS activity *p*-value = 4 × 10^−3^, glycolytic activity *p*-value = 2 × 10^−7^, Wilcoxon rank-sum tests were applied with significance threshold of 0.05 for DEG discovery, and Benjamini-Hochberg procedure was used to obtain multiple-testing corrected *p*-values (3% O_2_: 96 cells; 20% O_2_: 85 cells; *n* = 3). **c** Line plot showing the number of mtDNA-replication-related genes expressed per cell (mtDNA replication score, *x* axis) during PGCLC differentiation using the conventional protocol (red, 180 cells, *n* = 3) and modified protocol (blue, 184 cells, *n* = 3). The mtDNA replication score is calculated based on the expression of 18 genes (Supplementary Data 2), *p*-value = 0.0004, Wilcoxon rank-sum tests were applied with significance threshold of 0.05 for DEG discovery, and Benjamini-Hochberg procedure was used to obtain multiple-testing corrected *p*-values. **d**, **e** *Mgme1* and *Mthfd2* gene expressions during PGCLC differentiation at 20% (**d**) and 3% (**e**) oxygen concentration. Data represent the median of read counts ± SD. Wilcoxon rank-sum tests were applied with significance threshold of 0.05 for Differentially Expressed Gene (DEG) discovery and Benjamini-Hochberg procedure was used to obtain multiple-testing corrected *p*-values.

First, we analysed the expression of lineage-specific genes^12^ in cells isolated at D7 (Fig. S8a). Unbiased hierarchical clustering did not distinguish *BVSC*(+) cells generated using the conventional (20% oxygen) protocol from the modified (3% oxygen) protocol. This indicates that, under both conditions, we generated near-identical PGCLCs from ESCs in vitro. Next, we identified genes that were differentially expressed between high- and low-oxygen conditions from D4 to D7 (Fig. 4a). The transcriptomes diverged the most at D5 and D6 (Fig. 4a), which also correspond to the lowest levels of mtDNA (Fig. 1g). The in vitro genetic bottleneck occurred alongside the metabolic shift from a predominant OXPHOS-dependent to a more balanced metabolism (significant glycolytic activity increase with *p*-value = 2 × 10^−7^; Fig. 4b and Supplementary Data 2), closely resembling the metabolic profile of cells at E7.5 in vivo^27^, and corresponding to the in vivo mtDNA bottleneck^10^ (Figs. 1e and S2a).

Next, we focussed on genes involved in the mtDNA replication, calculating a score reflecting the number of mtDNA-replication-related genes (Supplementary Data 2) expressed per cell on each day of in vitro development. Less replication genes were expressed in cells under low-oxygen conditions than those under high-oxygen conditions between D5 and D7 (Fig. 4c, *p*-value = 0.0004), and the number of genes expressed at D5 was significantly lower at 3% oxygen (Fig. S8b; *p*-value = 0.011). Similarly, *BVSC*(−) cells displayed lower expression of mtDNA-replication-related genes in comparison to *BVSC*(+) (*p*-value = 7.5 × 10^−22^, Fig. S8c). These data support our earlier observations, implicating a reduction of mtDNA replication in generating the mtDNA genetic bottleneck. To illustrate this, we compared two specific genes: (1) the mitochondrial genome maintenance exonuclease 1 (*Mgme1*) shown to be involved in the degradation of linear double-stranded mtDNA during replication^34^ and (2) the mitochondrial methylenetetrahydrofolate dehydrogenases (*Mthfd2*), member of the folate-mediated one-carbon metabolism that supports mtDNA replication via the nucleotide synthesis within the organelle^35^. At 20% O_2_, these two genes appeared to be co-expressed throughout the differentiation process (Fig. 4d) suggesting a balance between mtDNA degradation and replication in line with a constant mtDNA content as well as number of mtDNA-replicating foci. However, at low oxygen concentration, *Mgme1* expression was significantly increased from D4 to D5 (Figs. 4e and S8d, *p*-value = 0.0012). *Mthfd2* level remained constant from D4 to D5 with a significantly reduced expression when compared to 20% O_2_ at D5 (Fig. S8d, *p*-value = 0.0034), implying a disruption of the balance between mtDNA degradation and replication (Fig. 4e) potentially contributing to the drop in mtDNA copy number. In line with this, at D7 after differentiation using the modified protocol, the expression of these two genes is once again balanced out (Fig. 4e). Indeed, we observed a significant increase in *Mthfd2* expression from D6 to D7 (Figs. 4e and S8d, *p*-value = 0.016) combined with a reduction of *Mgme1* expression in low compared to high oxygen concentrations (Fig. S8d, *p*-value = 0.037), correlating with the increase of mtDNA copy number observed at this stage (Fig. 1g). Taken together, this supports a role for mtDNA replication as an important mechanism in the generation of the mtDNA genetic bottleneck, and it also illustrates the complexity of its regulation.

Finally, we investigated the expression of genes involved in mitochondrial dynamics at D5 of differentiation. These pointed towards a more fragmented mitochondrial network at low oxygen concentrations, which correlated with the decreased mtDNA content, particularly involving *Drp1*, *Yme1l1* and *Bnip3* (Fig. S9a). Nonetheless and in keeping with our previous observations (Fig. 2b), there was no difference in autophagy-related gene expression between the conventional and modified protocol at D5 (Fig. S9b and Supplementary Data 2), and similar expression levels for genes linked to lysosomal activity throughout the differentiation process (Fig. S9c and Supplementary Data 2). Thus, the RNA-seq data support our earlier conclusion that autophagy are not primary mechanisms generating the mtDNA genetic bottleneck.

## Discussion

In several vertebrate species, the amount of mtDNA falls to low levels in the developing female germ line shortly after implantation of the blastocyst^7,8,10,11^. In silico simulations and statistical modelling suggest that the reduction in mtDNA content is sufficient to contribute to a genetic bottleneck leading to an increase in the heteroplasmy variance in the oocyte population^13^, but there is limited direct experimental evidence to support this.

Here we show that heteroplasmy segregation of the *mt-Nd1* variant occurs only under the conditions when mtDNA levels are low. The observed reduction in mtDNA content at 3% oxygen levels closely resembles the mtDNA genetic bottleneck measured in single cells in mice in vivo^10–12^, and suggests that low oxygen tension contributes to the formation of a mtDNA genetic bottleneck. In vivo measurements of the mtDNA bottleneck have only been made on committed PGCs, defined by alkaline phosphatase staining, or *Stella* (SC) expression. However, the in vitro model allows the isolation of pre-PGCLCs based on *BV*(+) expression, which is not technically possible in vivo. The mtDNA levels were lowest in the *BV*(+) cells, indicating that the lowest cellular mtDNA content (or the ‘narrowest’ part of the bottleneck), is actually immediately before the PGCLC stage. Experimentally raising the oxygen concentration prevented the reduction in mtDNA content, and abolished heteroplasmy segregation in PGCLCs confirming the importance of local oxygen levels, and showing that the reduction in mtDNA content is required for the genetic bottleneck mechanism. Although it is possible that the PGCLCs we generated at 3% oxygen were not developmentally competent, we think this is highly unlikely because they were generated at the same frequency without showing signs of stress (autophagy and cell viability), had near-identical expression profiles for key developmental genes and expressed the same lineage-specific cell markers. It therefore seems highly likely that changes in the local cellular micro-environment during and after implantation are playing a key role in driving the mtDNA genetic bottleneck itself, and thus is important for the segregation of mtDNA heteroplasmy.

Establishing an in vitro model has also cast light on sub-cellular mechanisms contributing to the bottleneck. In ESCs, mtDNA levels remained stable at ~1100 copies despite ongoing cell division over 9 passages, consistent with an active control mechanism regulating the intracellular mtDNA content (Fig. S1i). However, the induction of differentiation led to a reduction in mtDNA levels regardless of the cell proliferation rates, associated with a reduction in the number of mtDNA-replicating foci per cell. Although, based on our data, the reduction in replicating foci cannot completely account for the mtDNA bottleneck, sc-RNA-seq data independently endorse the importance of mtDNA replication in the bottleneck mechanism, and points towards an active mechanism reducing the intracellular mtDNA content. Finally, given that each mtDNA nucleoid is thought to contain ~1.4 mtDNA molecules, only a minority of the mtDNA nucleoids were actively replicating at any one time. It is therefore possible that this ‘sub-sampling’ also contributes to the overall bottleneck mechanism in vivo^11^, explaining why simple models based only on total-cell mtDNA levels only account for ~70% variance in heteroplasmy seen in the next generation^10^.

It is striking that we observed an important range of mtDNA copy number per cell at all stages of the differentiation. These differences could reflect the precise developmental stage of each cell^36,37^, which is unlikely to be completely synchronised, natural fluctuations in mtDNA content accompanying the cell cycle^38^, or conceivably, local differences in oxygen concentration. Recent evidence has shown heterogeneity in the oxygen concentration within individual cultured cells^39,40^. This is also likely to be the case in our experiments, potentially explaining why *BV*(+), *BVSC*(+) and *BVSC*(−) cells showed a wide range in their mtDNA content at every developmental stage. Similar cell heterogeneity is also likely in vivo, also explaining the range of mtDNA levels seen in PGCs and somatic cells^10,11^. Although this means that we cannot make a definitive statement about a specific oxygen concentration that is required to cause the mtDNA bottleneck, our main conclusion—that oxygen levels modulate the mtDNA bottleneck—still holds true. Different pathogenic mtDNA mutations can also influence the cellular oxygen consumption to differing degrees, and thus potentially influence the cellular micro-environment when oxygen is limited. It is therefore plausible that the mutations themselves alter the intracellular mtDNA content, as seen in blood cells^41^. If this also occurs within PGCs at a critical stage of development in vivo, it would lead to mtDNA bottlenecks of different ‘strengths’, as seen in families transmitting pathogenic mtDNA mutations where m.8993T > G segregates more rapidly than other pathogenic mtDNA mutations^42^. It will therefore be important to study other mtDNA variants in our in vitro system, to determine whether they behave in a similar way to the *mt-Nd1* variant we studied here.

Mutation-specific heteroplasmy segregation has also been observed in mice^43,44^, and in *Drosophila*, where specific variants undergo ‘selfish’ propagation during oogenesis^45^, or undergo purifying selection linked to mitochondrial fragmentation and mitophagy/autophagy^46^. In our in vitro model, *BVSC*(+) cells showed high ATG12 levels, but we saw no evidence of selection against the *mt-Nd1* variant at that time point. On the other hand, although we did see the signature of selection in *BVSC*(−) cells, this was not associated with ATG12 activation, nor any evidence of transcriptional activation of autophagy mechanisms (Fig. S9d, *p*-value = 0.0005). Taken together, these findings indicate that ATG12-mediated autophagy is unlikely to be involved in selection at this stage of development. However, it is important to note that our observations do not exclude the possibility that ATG12 is involved in selection at a later stage, or that there is an ATG12-independent mechanism as previously proposed^46^. The selection in *BVSC*(−) cells could be occurring at several levels: at the mtDNA level, at the organelle level or at the cellular level. The absence of ATG12 activation in the ND1 *BVSC*(−) cells suggests that the selection is not occurring at the sub-cellular level. However, the mitochondrial polarisation we observed could contribute to the selection through the unequal partitioning of heteroplasmy during cell division. It is also possible that differences in cell proliferation are involved. In keeping with this, we noted that *BVSC*(−) cells proliferated faster than the *BVSC*(+) cells. If *BVSC*(−) cells containing high levels of the ND1 variant proliferated slower due to the mild defect of mitochondrial respiration, this would contribute to a reduction in the average mutation load. The signature of selection that we observed in *BVSC*(−) cells contrasts with findings in human embryos^12^, where selection was observed in PGCs but not in adjacent somatic tissues. However, Floros et al.^12^ studied low-level mtDNA heteroplasmies across the whole mitochondrial genome, and focussed on a later stage of post-implantation development. Different selection mechanisms are known to act at different points during transmission^44,47^, potentially explaining these differences and highlighting the complexity of heteroplasmy inheritance mechanisms, which may be mutation dependant.

Our observations show that oxygen is likely to make an important contribution to the mtDNA bottleneck. Validating this in vivo could open up new approaches to influence heteroplasmy levels in somatic cells and within the germ line, and thus open new therapeutic avenues to treat and prevent mtDNA diseases.

## Methods

### Embryonic stem cell maintenance and coating procedures

ESCs were cultured in a 2i + LIF medium as previously described^21^. Briefly, ESCs were cultured in poly-L-ornithine (0.01%; Sigma) and laminin (10 ng/ml; Sigma) pre-coated 6-well plates in N2B27 medium supplemented with 2i (CHIR99021, 3 μM: Stemgent; PD0325901, 0.4 μM: Stemgent) and LIF (10^3^ units/ml; Millipore). The expended colonies were passaged every other day by TrypLE (Invitrogen) dissociation. Plate coating was performed for 1 h at room temperature (RT) with poly-L-ornithine and for 1 h at 37 °C with laminin. Plates were washed with PBS (Invitrogen) before cell plating.

### DNA extraction from single cells

Single ESCs were sorted in 96-well plate and lysed for 30 min at 37 °C in 4 µl of 50 mM Tris-HCL, pH 8.5, with 0.5% Tween20 and 200 ng/ml proteinase K (Ambion) followed by 15 min of inactivation by heating at 80 °C.

### Deep sequencing and variant analysis

mtDNA was amplified using PrimeSTAR GXL DNA polymerase (Takara) in two overlapping fragments (Amplicon 1, forward primer: AGCAAAAGCCCACTTCGCCA, reverse primer GGTTGGCCCCCAATTCAGGT; Amplicon 2, forward primer: ACCTGAATTGGGGGCCAACC, reverse primer TGGCGAAGTGGGCTTTTGCT)^48^. PCR products were assessed by gel electrophoresis and each amplicon was purified using Agencourt AMPure XP beads (Beckman-coulter), quantified with a Qubit 2.0 fluorimeter (Invitrogen) and equal concentrations of amplicon 1 and amplicon 2 from the same single cell were pulled together to obtain a final concentration of 0.2 ng/µl. Library preparation was performed using the Nextera DNA preparation kit (Illumina). Pooled amplicons were tagmented, amplified, cleaned and pooled in equimolar concentrations. The library was sequenced with a Mi-Seq Reagent Kit v2 for 600 cycles (Illumina) in paired-end, 251 bp reads. Post-run FASTQ files were analysed using an in-house-developed bioinformatics pipeline. Reads were aligned to the C57Bl6/J mouse reference sequence using BWA^49^. Aligned reads were sorted and indexed using Samtools^50^ and duplicated reads were removed using Picard (http://broadinstitute.github.io/picard). Variant calling was performed in tandem using VarScan2^51^. Variants were annotated using ANNOVAR^52^ and heteroplasmic variants were defined as >1% minor allele frequency.

### Embryonic stem cell subcloning

Cells were seeded at low confluency (10,000 cells per well) and single cells were picked using a mouth pipette under a Nikon SMZ1000 microscope before to be transferred into a gelatin (0.1%)-coated 24-well plate. Single cells were incubated at 5% CO_2_, 37 °C until ESC colony was visible under microscope. The ESC colony was dissociated with trypsin and reseeded into a 24-well plate coated with gelatin and incubated for 48 h at 37 °C with 5% CO_2_ before to be expanded and cultured as described in section ‘Embryonic stem cell maintenance and coating procedures’.

### Induction of EpiLCs and PGCLCs

Epiblast-like cell differentiation from ESCs and PGC-like cell induction from EpiLCs were performed as described previously unless specified otherwise^21^.

### Fluorescence-activated cell sorting (FACS)

FACS was monitored by the staff of the cytometry facility at the Cambridge Institute for Medical Research (CIMR) (University of Cambridge). Cell sorting was done using a high-speed influx cell sorter (BD Biosciences) that has four lasers at 405, 488, 561 and 640 nm and is equipped with 16 fluorescence detectors and a small particle detector. ESCs were trypsinized, centrifuged and resuspended in 2i + LIF medium. The cell suspension was filtered using CellTrics 50 µm (Sysmex) in round-bottom polystyrene tubes (Falcon). Tubes were kept on ice for DNA analysis purposes and at RT for immunofluorescence (IF). Embryonic bodies were collected at various days of differentiation and transferred to a 15 ml Falcon tube and centrifuged for 1 min at 200*g*. The supernatant was transferred to a new 15 ml Falcon tube and kept at 37 °C. Embryonic bodies were resuspended with 500 μl of trypsin supplemented with ethylenediaminetetraacetic acid (Trypsin-EDTA) and incubated at 37 °C for 4, 7, 8 and 10 min at day 2, 3, 4 and 5 of the differentiation, respectively. The enzyme activity was blocked by adding 500 μl of GMEM. The cell suspension was centrifuged for 5 min at 200*g* at RT. The supernatant was aspirated. The pellet was resuspended with the PGCLC medium kept at 37 °C and previously filtered. Cells used to investigate the number of replicative mtDNA molecules were incubated for 1 h at 37 °C with EDU (1/1000). After incubation, cells were centrifuged and resuspended in PGCLC medium. The cell suspensions were filtered using CellTrics 50 μM (Sysmex) in round-bottom polystyrene tubes (Falcon). Draq7 (1/1000; Abcam) was added to each tube to assess cell viability. Tubes were kept on ice for DNA analysis purposes and at RT for IF. Fluorescence intensity in the YFP channel was plotted against the fluorescence intensity in the CFP channel^21^. Single cells sorted in 96-well plates for DNA analysis were immediately put on dry ice before to be stored at −80 °C. Cells used for immunochemistry were sorted by FACS (between 200 and 500 cells) on coverslips coated with gelatin (0.1%) in a 24-well plate. Cells were incubated in the plate for 30 min at 37 °C in either 20% or 3% of oxygen for cell adhesion. Cells for cell cycle analysis were incubated with 2 µM Hoechst. Gates were designed such that G1 and G2/M contain 5% of less positive cells and 5% of the more positive cells for Hoechst intensity, respectively.

### Mitochondrial DNA copy number measurements

Quantitative real-time PCR (qPCR) was performed on a CFX96 Touch Real-Time PCR detection system (Bio-Rad). mtDNA copy number was calculated using a Taqman assay targeting the *MT-ND5* gene (Forward primer: ACCTAATTAAACACATCAACTTCCC; Reverse primer: GACTCAGTGCCAGGTTGTAA; Probe: HEX-ATTGCCTTTCTGACTAGGTG-BHQ_1) and the *β-actin* gene (Forward primer: GATCGATGCCGGTGCTAAGA; Reverse primer: GGAAAAGAGCCTCAGGGCAT; Probe: FAM- ACACCACCACATCAATCAAATTCTCCTTCA -BHQ_1). PCR-generated templates were used to generate standard curve and make absolute quantification. Single-cell mtDNA copy number measurements were carried out by Droplet Digital PCR (ddPCR) according to the manufacturer’s instructions, using two mitochondrial target *mt-Nd1* (Forward primer: GAGCCTCAAACTCCAAATACTCACT; reverse primer: GAACTGATAAAAGGATAATAGCTATGGTTACTTCA; probe sequence: FAM-CCGTAGCCCAAACAAT-BHQ_1) and *mt-Co3* (Forward primer: CCTCGTACCAACACATGATCTAGG; reverse primer: AGTGGGACTTCTAGAGGGTTAAGTG; probe sequence: HEX - ACCTCCAACAGGAATTTCA-BHQ_1)^53^. Droplet analysis was performed using the QuantaSoft analysis software (Bio-Rad). The absolute mtDNA copy number represents an average of the positive droplets (HEX/FAM).

### Western blotting

After migration, proteins were transferred to a polyvinylidene difluoride membrane with the iBlot 2 Semi Dry Transfer system (Thermo Fisher Scientific). iBlot2 protocol P0 (20 V for 1 min, 23 V for 4 min and 25 V for remainder) was used for 7 min. Membranes were washed in Tris Buffer Saline with Tween 20 (TBST) buffer made of 20 mM Tris hydrochloride pH 7 (Sigma-Aldrich), 0.5 mM NaCl (VWR, Lutterworth, UK) and 0.1% Tween 20 (Sigma-Aldrich)), and then blocked with 5% (w/v) milk in TBST for 30–60 min. Primary antibodies were incubated overnight at 4 °C (mt-Co1, ab14705, 1/1000; β-*actin*, a1978, 1/5000; Mito-Cocktail, ab110413, 1/1000). The membrane was washed in TBST and incubated with a secondary antibody for 1 h at RT. The protein signal was developed with Clarity Western ECL Western Blotting substrate (Bio-Rad) for 5 min in the dark and imaged using the Amersham Imager 600 (GE Healthcare). Densitometric analysis of the protein signal was performed using ImageQuant TL 8.1 software (GE Healthcare) and a ratio of the results, Protein constitutively expressed/Protein of interest was calculated.

### Mitochondrial respiration measurements

Mitochondrial oxygen consumption rate (OCR) of WT and ND1 ESCs was analysed with SeahorseXFe24 Analyzer. Final concentration of 1 µM Oligomycin, 1.5 µM FCCP and 1 µM Rotenone/1 µM antimycin A was applied sequentially. After extracellular flux analysis, cells were lysed in 20 µM lysis buffer and used for Bradford protein assay. Seahorse data were normalised protein level.

### Pyrosequencing

PCR primers and the sequencing primers were generated by the PyroMark Assay Design Software (Qiagen). Forward primer: TCTATGAGTTCCCCTACCAATACC; Biotinylated reverse primer: AAATTGTTTGGGCTACGGC; Sequencing primer: TTTAAACCTAGGGATTTTAT^54^. Templates for pyrosequencing were amplified using the PyroMark amplification PCR kit (Qiagen) using the manufacturer’s instructions. PCR amplification was programmed as follows: 95 °C for 15 min as initial PCR activation step, then 45 cycles at 94 °C for 30 s for denaturation, 60 °C for 45 s for annealing and 72 °C for 30 s for extension. A final extension step of 10 min at 72 °C clotures the PCR programme before the samples were stored at 4 °C. Sequencing of the PCR amplicons was performed using a PyroMark Q48 instrument (Qiagen) according to the manufacturer’s instructions. Sequence analysis was performed using the PyroMark Supplementary Software.

### Immunocytochemistry

Cells were fixed with 4% paraformaldehyde (PFA) for 15 min at 37 °C in either 20% or 3% of oxygen. PFA was washed 3 times with 5% foetal bovine serum (FBS)/PBS. PFA was quenched with a 10 mM NH_4_Cl/PBS for 10 min at RT in a humid chamber. NH_4_Cl/PBS was washed 3 times with 5% FBS/PBS. Cells were permeabilized with 0.1X-Triton for 10 min at RT in a humid chamber. Then, 0.1X-Triton was washed 3 times with 5% FBS/PBS. To investigate the mtDNA replication, Click-iT EDU imaging kit was used following manufacturer’s instructions. The Click-iT reaction cocktail was prepared with Click-iT reaction buffer, copper protectant, Alexa Fluorpicolyl Azid and reaction buffer additive. The cocktail was incubated on a coverslip for 30 min at RT in a dark, humid chamber. Cocktail was washed 3 times with 5% FBS/PBS^29^. Coverslips were blocked for 20 min at RT in a dark, humid chamber. Cells were directly incubated for 1 h at RT in a dark, humid chamber with rabbit anti-TOM20 (1:200 Abcam), mouse anti-TOM20 (1:200 Abcam), mouse anti-ATP5a (1:200 Abcam) and rabbit anti-ATG12 (1:200 Cell Signalling) prepared in 5% FBS/PBS. Coverslips were washed 3 times with 5% FBS/PBS. TOM20 was revealed with a donkey anti-rabbit Alexa 594 or anti-mouse Alexa 488 (1:1000), goat anti-mouse Alexa 594 (1:1000) and a goat anti-rabbit Alexa 647 (1:1000). Secondary antibodies were incubated for 1 h at RT in a dark, humid chamber before to be washed 3 times with PBS. Coverslips were washed with water and mounted on slides using a fluorescent mounting media (Dako)^55^.

### Confocal scanning imaging

Images of the Embryonic Bodies were acquired with a LSM880 Axio Observer scanning system (Zeiss) using the lasers 458 nm (for CFP) and 514 nm (for YFP) and the 10x/0.3 EC Plan Neofluar dry objective. All images were acquired using the same parameters across the differentiation.

### Confocal spinning disk imaging

Cell imaging was performed on an Andor Dragonfly 500 confocal spinning disk system (Andor) using a Zyla 4.2 Megapixel PLUS sCMOS camera (Andor). Fusion was used as the acquisition software provided by Andor. Here, 561 nm (50% intensity) and 637 nm (80% intensity) laser were used to acquire the images with the Nikon 100X/NA1.4 oil immersion objective of the Nikon Eclipse TiE inverted microscope. The same parameters of laser intensity and exposure time (500 ms) were used for all conditions across the differentiation. The whole cell was acquired using a Piezo Z-stage (0.2 μm stack); 3D reconstituted images were deconvolved using Fusion deconvolution algorithm to reassign out-of-focus light contribution to their initial location in the source image.

### Image processing

The 3D reconstituted images were analysed using IMARIS software (Bitplane). The intensity of each signal was set with a different threshold for each day of the differentiation but remained the same across conditions. Surfaces were created for TOM20, ATP5a and ATG12 signals with a smooth surface detail of 0.001, 0.05 and 0.05 μm, respectively. The threshold of detection was adjusted manually to ensure the intensity detection made by the software corresponds to the signal identified by eye. A filter was applied to remove unspecific created surfaces below 0.5 µm for TOM20 and ATP5a signals. The bottom half of cells was removed from the analysis and a filter was created for ATG12 signal to remove the background. To identify and count the number of replicating mtDNA molecules, a spot detection was created to detect the Alexa 647 intensity signal with similar parameters as the surfaces mentioned above. Spots were assigned to an intensity signal with an estimated diameter of 0.6 μm. The number of spots colocalizing with the mitochondria was calculated by IMARIS using a ‘distance surface close to spot’ tool with a threshold of distance equal to 0. Data were normalised to the same untreated control cell line prepared and analysed in parallel on each day to account for the day-to-day experimental variability. For polarisation analysis, a spot surface was generated for TOM20 intensity. A reference point was placed at the centre of the cell in its 3D space allowing the division of each cell into 8 sections according to the *x*, *y* and *z* axes. Spots present in each section were counted and divided by the total number of count per cell (p_i). The polarity index was calculated as the entropy of all 8 p_i.

### sc-RNA-seq and gene expression analysis

Single cells were sorted by FACS into the wells of a 96-well plate containing lysis buffer. Illumina Nextera XT DNA preparation kit was used to prepare the libraries. Pooled libraries of 96 cells were sequenced on a Hi-Seq 2500 (single-end 50 bp read length). Raw FASTQ files from 10x sequencing set were demultiplexed and aligned to the GRCm39 reference genome using STARsolo^56^, with default settings set according to the 10x chromium chemistry used. For the expression matrix analysis, we used Scanpy and performed standard pre-processing steps: removing cells with less than 200 genes, removing genes that have been detected in less than 3 cells, normalisation to 10,000 reads per cell and log-transformation^57^. Wilcoxon rank-sum tests were used with significance threshold of 0.05 for DEG discovery and use the Benjamini-Hochberg procedure to obtain multiple-testing corrected *p*-values. To assign each cell an OXPHOS, glycolytic, autophagy and mtDNA replication scores, we count the number of genes that are expressed in each cell, which are annotated within their respective gene list (Supplementary Data 2) or with their respective GO term list (GO:0006096 for ‘glycolytic process’ and GO:0006119 for ‘oxidative phosphorylation’) and normalise the score.

### Reporting summary

Further information on research design is available in the Nature Research Reporting Summary linked to this article.

## Supplementary information

Supplementary information.

Description of Additional Supplementary Files.

Supplementary Video 1.

Supplementary Data 1.

Supplementary Data 2.

Supplementary Data 3.

Reporting summary.

## Data Availability

Raw sequencing data are available in the Gene Expression Omnibus under accession number GSE169427. Raw data corresponding to the figures are included in Supplementary Data 1, and heteroplasmy data are shown in Supplementary Data 3. Any remaining information can be obtained from the corresponding author upon reasonable request.
