# Supplementary material for: Oxygen tension modulates the mitochondrial genetic bottleneck and influences the segregation of a heteroplasmic mtDNA variant in vitro
Source: Commun Biol. 2021 May 14;4:584. doi: 10.1038/s42003-021-02069-2 (PMC8121860; doi:10.1038/s42003-021-02069-2)
Supplement: Supplementary file 1 — Supplementary information. [file 42003_2021_2069_MOESM1_ESM.pdf]

Supplementary Information

Oxygen tension modulates the mitochondrial genetic bottleneck and influences the segregation of a heteroplasmic mtDNA variant

<u>Content:</u> Supplementary figure (1 : 9)

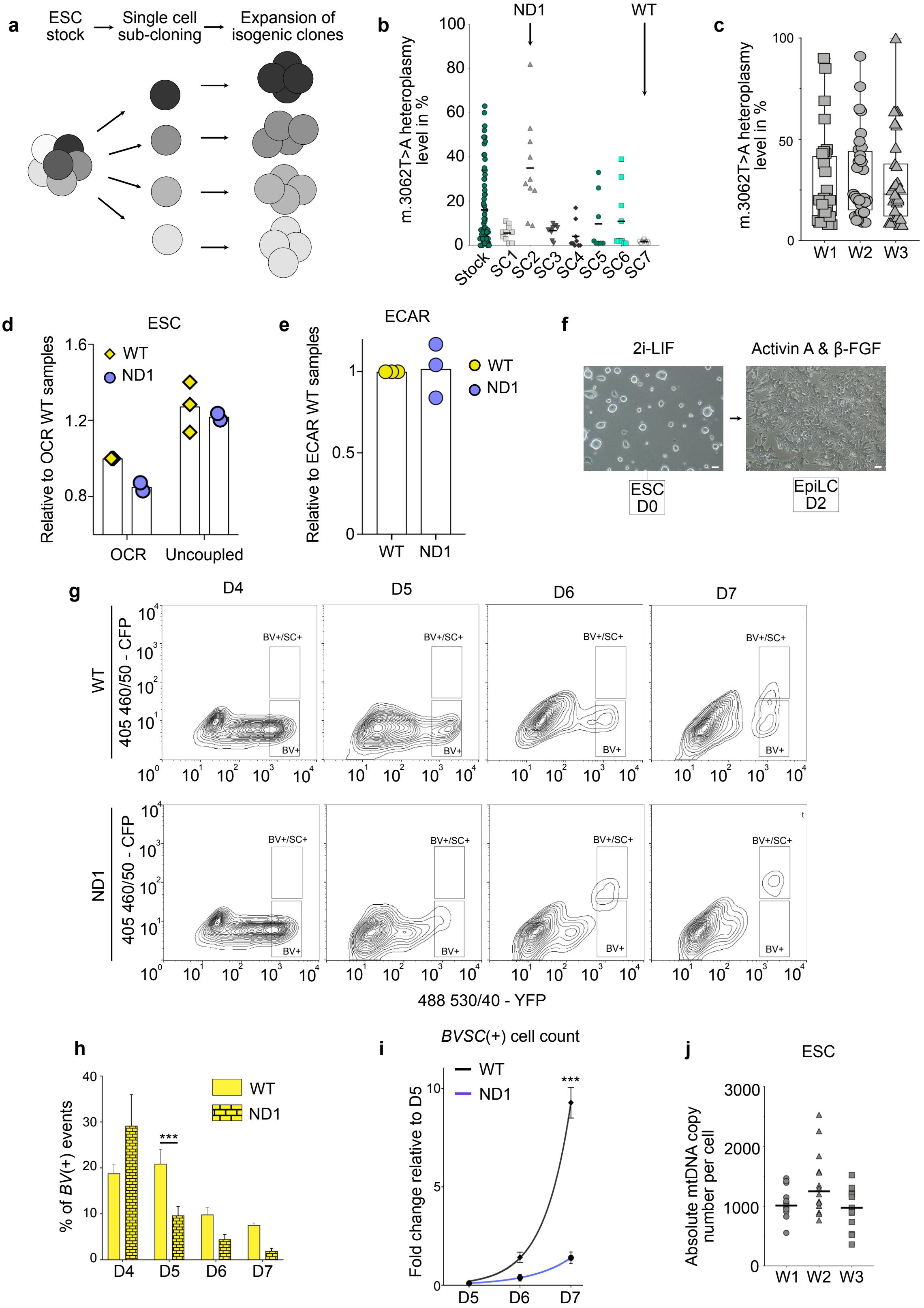

a
ESC stock
Single cell sub-cloning
Expansion of isogenic clones
b
ND1
WT
m.3062T>A heteroplasmy level in %
Stock
SC1
SC2
SC3
SC4
SC5
SC6
SC7
c
m.3062T>A heteroplasmy level in %
W1
W2
W3
d
ESC
WT
ND1
Relative to OCR WT samples
OCR
Uncoupled
e
ECAR
WT
ND1
Relative to ECAR WT samples
f
2i-LIF
Activin A & β-FGF
ESC D0
EpiLC D2
g
D4
D5
D6
D7
WT
ND1
405 460/50 - CFP
BV+/SC+
BV+
488 530/40 - YFP
h
WT
ND1
% of BV(+) events
i
BVSC(+) cell count
WT
ND1
Fold change relative to D5
j
ESC
Absolute mtDNA copy number per cell

Supplementary Figure 1: Differentiation of primordial germ cell-like cells (PGCLCs) *in vitro* in 20% oxygen. a, Scheme highlighting the sub-cloning method to obtain isogenic clones. **b**, Heteroplasmy measurements of m.3062T>A:p.mt-nd1 per cell in embryonic stem cells (ESCs) before subcloning (Stock) and after subcloning (SC: Sub-Clones). SC2 and SC7 were defined as ND1 and WT sub-clones respectively (n=10 cells per sub-clone). **c**, Heteroplasmy measurements of m.3062T>A:p.mt-nd1 per cell in ESC (W1: week number 1, 32 cells, n=2; W2: week number 2, 32 cells, n=2, W3: week number 3, 31 cells, n=2)**. d**, Mitochondrial oxygen consumption rates (OCR) in WT and ND1 ESCs. **e**, Measurement of the extracellular acidification rate (ECAR) using WT and ND1 ESCs. **f**, Bright field microscopy images of ESC and Epiblast cells. Scale bar = 300 nm. **g**, Representative example of contour plots of FACS, highlighted boxes in the plots show the selected cell population. **h**, Percentages of the *BV* (+) events acquired by FACS (mean +/- SEM, n=4 independent differentiation, ***p<0.001, two-way ANOVA with multiple comparisons using Sidak test). **i**, Proliferation rate of WT and ND1 *BVSC*(+) cells during differentiation to PGCLC at 20% O_2. Cell counts normalized to D5, data represent mean ± SEM, n=3, ***p<0.001, Student t-test. **j**, Absolute mtDNA copy number measurement per single ESCs measured over 3 weeks (5 passages total (W1: week number 1, n=14 cells; W2: week number 2, 12 cells, W3: week number 3, 13 cells).

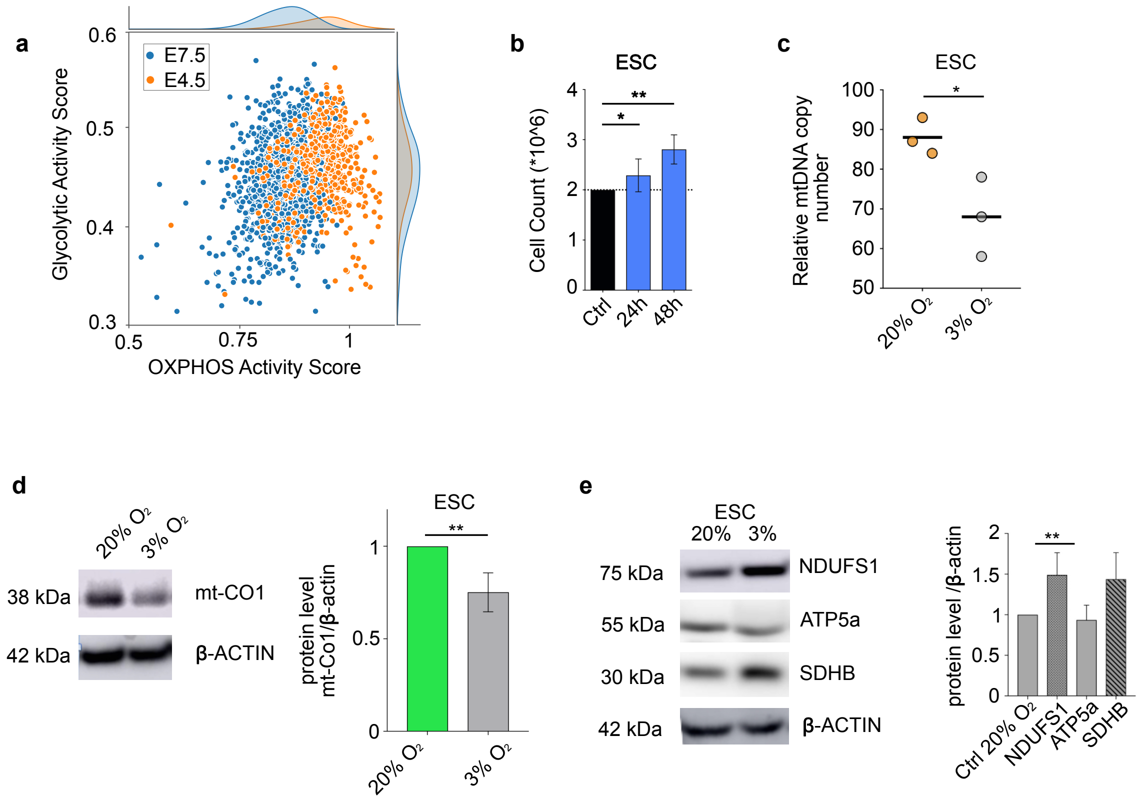

a
E7.5
E4.5
Glycolytic Activity Score
OXPHOS Activity Score
b
ESC
Cell Count (*10^6)
Ctrl
24h
48h
c
ESC
Relative mtDNA copy number
20% O2
3% O2
d
20% O2
3% O2
38 kDa
mt-CO1
42 kDa
β-ACTIN
ESC
protein level mt-Co1/β-actin
e
ESC
20%
3%
75 kDa
NDUFS1
55 kDa
ATP5a
30 kDa
SDHB
42 kDa
β-ACTIN
protein level /β-actin
Ctrl 20% O2
NDUFS1
ATP5a
SDHB

Supplementary Figure 2: Oxygen tension modulates mtDNA content in ESC. a, Scatter plots with marginal distributions showing the scRNA-seq analysis of metabolic genes expressed in cells collected from embryos at E4.5 and E7.5. Each dot represents a single cell. Its (x,y) coordinate is calculated by the number of genes expressed related to glycolytic (y axis) and OXPHOS (x axis) metabolism. Data consider the expression of 85 glycolytic genes and 96 OXPHOS genes (Supplementary table 2). OXPHOS activity p-value = 10^{-124}, Wilcoxon rank-sum tests were applied with significance threshold of 0.05 for DEG discovery and Benjamini–Hochberg procedure were used to obtain multiple-testing corrected p-values. **b**, Cell count of ESC maintained for 24 and 48h at 3% oxygen. The dotted line represents the number of ESC at 20% oxygen. Data represent the mean +/- SD, *p<0.05, **p<0.01 one-way ANOVA with multiple comparisons using Tukey test. **c**, Relative mtDNA copy number from bulk of DNA from ESC maintained at 20% O_2 or at 3% O_2 for 48h. The horizontal lines represent the mean of 3 independent experiments *p<0.05 (student t-test). **d**, mtDNA encoded protein levels in ESC maintained at 20% O_2 or at 3% O_2 for 48h. The level of mt-CO1 was normalised to β-actin level (n=3). **e**, Nuclear encoded mitochondrial protein levels in ESC maintained at 20% O_2 or at 3% O_2 for 48h. The level of protein was normalised to β-actin level, data represent the mean, **p<0.01, student t-test (n=3).

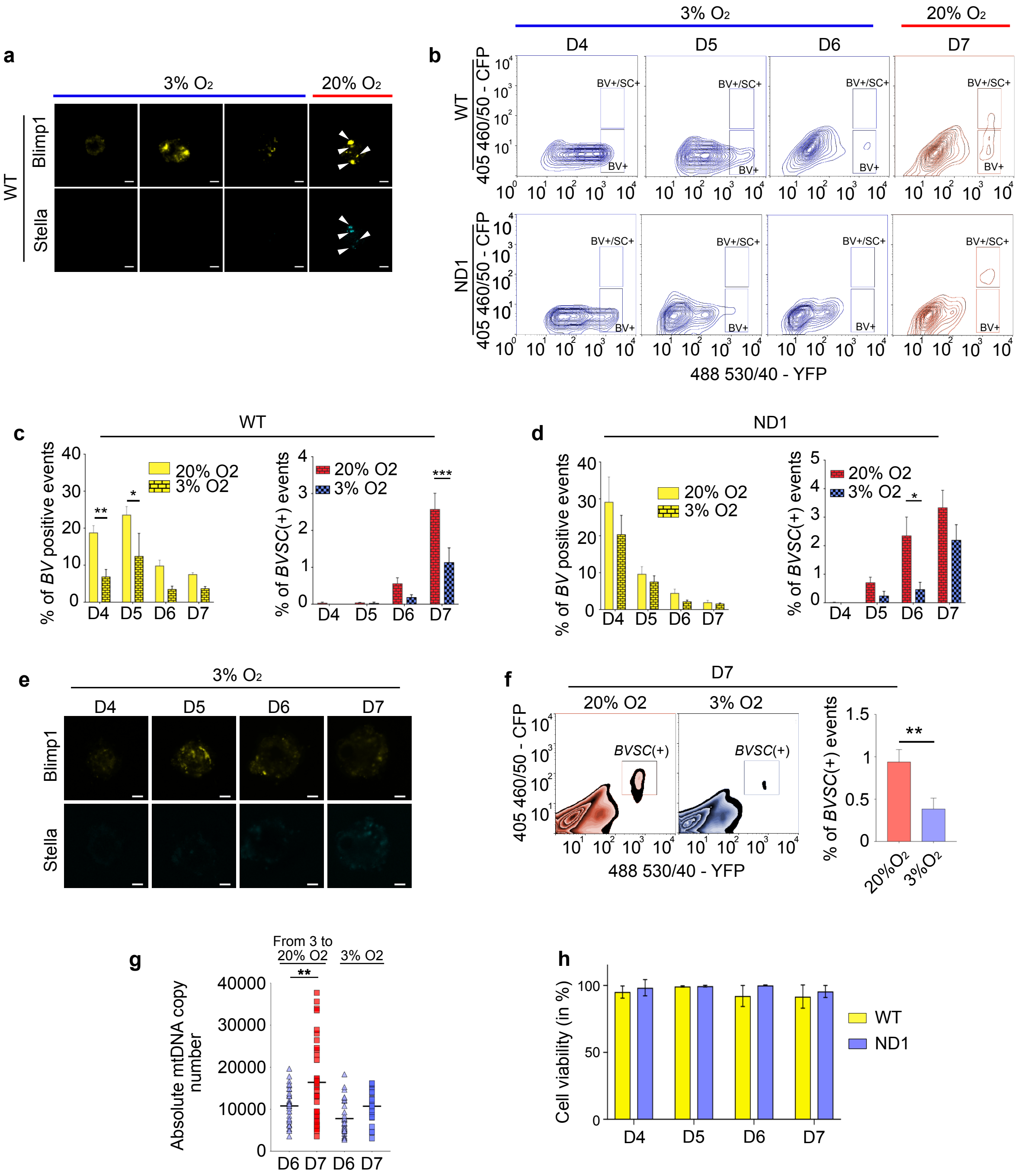
a
3% O2
20% O2
WT
Blimp1
Stella
b
3% O2
20% O2
D4
D5
D6
D7
WT
ND1
405 460/50 - CFP
BV+/SC+
BV+
488 530/40 - YFP
c
WT
% of BV positive events
20% O2
3% O2
% of BVSC(+) events
D4
D5
D6
D7
d
ND1
% of BV positive events
% of BVSC(+) events
e
3% O2
D4
D5
D6
D7
Blimp1
Stella
f
D7
20% O2
3% O2
405 460/50 - CFP
BVSC(+)
488 530/40 - YFP
% of BVSC(+) events
20%O2
3%O2
g
From 3 to 20% O2
3% O2
Absolute mtDNA copy number
D6
D7
h
Cell viability (in %)
WT
ND1

Supplementary Figure 3: Oxygen tension modulates PGC specific gene expressions. a, Monitoring of the differentiation of WT cells performed at various concentrations of oxygen. Representative microscopy confocal imaging. White arrows show PGCLCs. Scale bars = 100 µm. **b**, Representative contour plots (blue shades at 3% oxygen and red at 20% oxygen) of *BV* and *BVSC*(+) cells isolated by FACS (n=4). Highlighted boxes in the plots show the selected cell population. **c-d**, Percentages of the *BV*(+) and *BVSC*(+) events acquired from FACS using the conventional protocol of differentiation compared with the modified protocol of differentiation (mean +/- SEM, n=4, *p<0.05, **p<0.01, ***p<0.001, two-way ANOVA with multiple comparisons using Sidak test). **e**, Monitoring of the differentiation performed at low oxygen concentration up to D7. Representative microscopy confocal imaging. Scale bars = 100 µm. **f**, Representative FACS density plot of PGCLC at D7 differentiated in either 20 or 3% oxygen. Highlighted boxes in the plots show the selected cell population. Corresponding quantification of *BVSC*(+) events at D7 of differentiation are shown on the right panel. Data represent the mean ± SD from n=4 independent differentiations. **p<0.01 (student t-test). **g**, Absolute mtDNA copy number in cells during PGCLC differentiation from 3% oxygen (D6, light blue) to 20% oxygen (D7, red) concentration (left part of the graph) and in cell during PGCLC differentiation maintained at 3% oxygen (right part of the graph) from D6 (light blue) to D7 (dark blue). The horizontal lines represent the mean, *p<0.05 Two-way ANOVA with multiple comparisons using Sidak test (30 cells from 2 independent replicates). **h**, Effect caused by cell culture at low oxygen concentration on WT and ND1 cell viability in percentage, represented by the ratio of Drak7 negative cells at 20% oxygen and Drak7 negative cells at 3% oxygen. Data represent mean +/- SD (n=3).

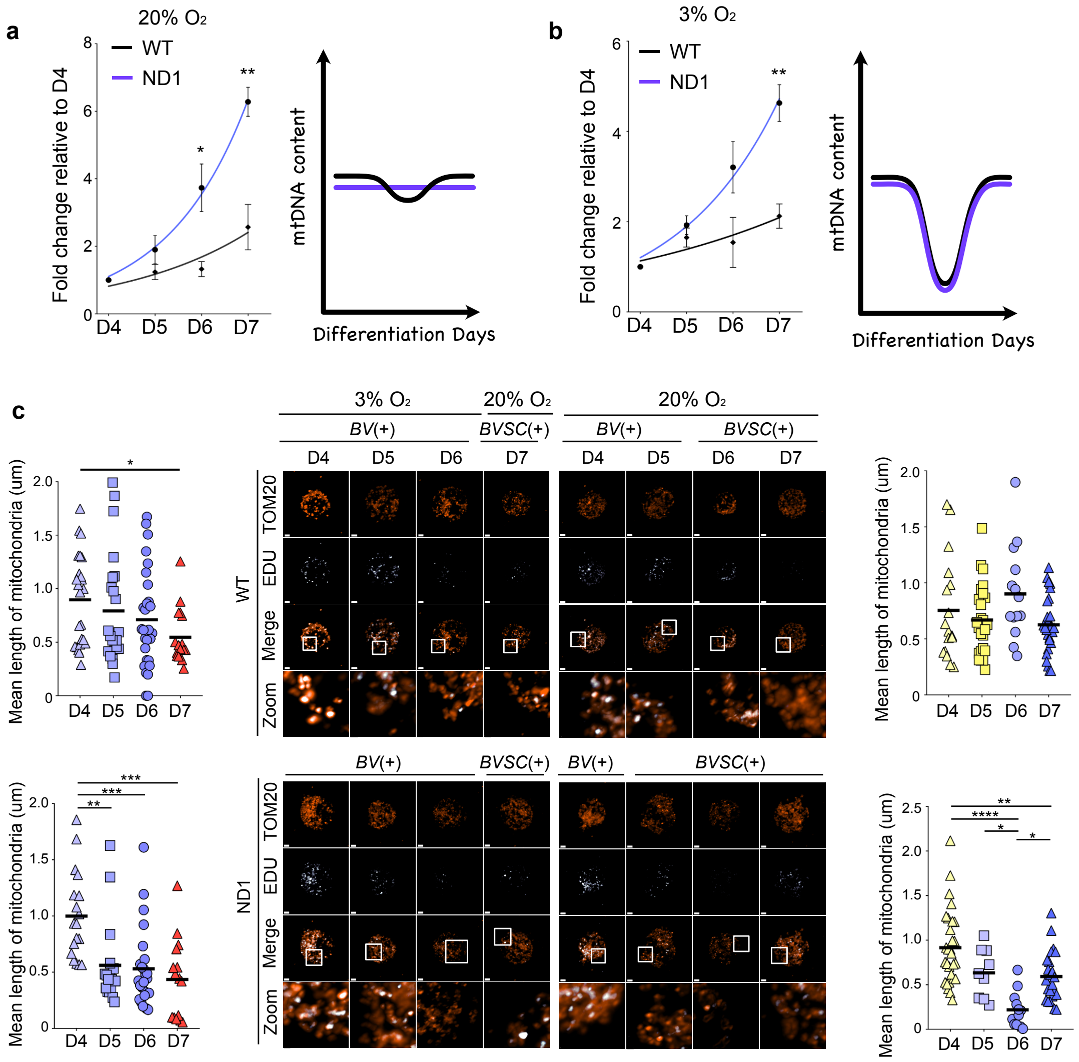
a
20% O2
WT
ND1
Fold change relative to D4
D4 D5 D6 D7
mtDNA content
Differentiation Days
b
3% O2
WT
ND1
Fold change relative to D4
D4 D5 D6 D7
mtDNA content
Differentiation Days
c
3% O2
20% O2
20% O2
BV(+)
BVSC(+)
BV(+)
BVSC(+)
D4 D5 D6 D7 D4 D5 D6 D7
WT
TOM20
EDU
Merge
Zoom
ND1
Mean length of mitochondria (um)
D4 D5 D6 D7

Supplementary Figure 4: Mitochondrial fragmentation correlates with low mtDNA content. **a**-**b**, Proliferation rate of WT and ND1 cells during differentiation to PGCLC at 20% O_2 (**a**) and 3% O_2 (**b**). Cell counts normalized to D4, data represent the mean ± SD, n=4, *p<0.05, **p<0.01, Student t-test). **c**, Representative microscopy confocal staining images of TOM20 intensity (red, upper) and labelled-pulsed with EDU spot (purple, lower) intensity signal of *BV* and *BVSC*(+) cells during PGCLC differentiation. Scale bars = 2 µm. Respective mean length of mitochondria quantifications are shown (colour code of the left panel reflects the concentration of oxygen; colour code of the right panel: reflects the expression of *BV*(yellow) and *BVSC*(blue) cells). Horizontal bars represent the mean (in µm), *p<0.05, **p<0.01, ***p<0.001 and ****p<0.0001, one-way ANOVA with multiple comparisons using Tukey test (WT - 3% O_2: D4: 25 cells, n=3; D5: 24 cells, n=3; D6: 26 cells, n=3; D7: 16 cells, n=2; ND1 - 3% O_2: D4: 19 cells, n=3; D5: 22 cells, n=3; D6: 24 cells, n=3; D7: 13 cells, n=2; (WT - 20% O_2: D4: 20 cells, n=3; D5: 25 cells, n=3; D6: 15 cells, n=3; D7: 27 cells, n=3; ND1 - 20% O_2: D4: 23 cells, n=3; D5: 10 cells, n=2; D6: 9 cells, n=2; D7: 20 cells, n=3).

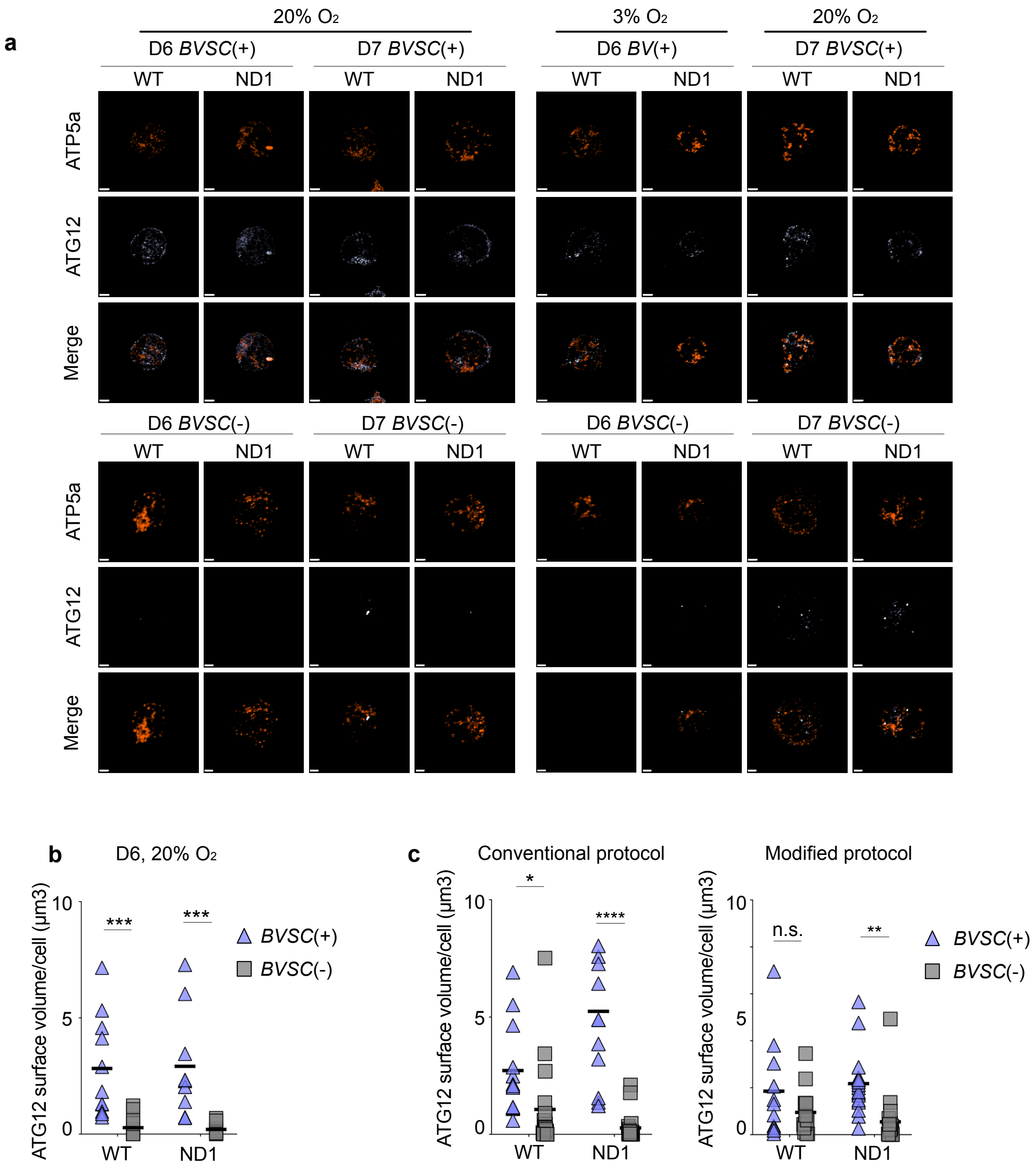

a
20% O2
D6 BVSC(+)
D7 BVSC(+)
3% O2
D6 BV(+)
20% O2
D7 BVSC(+)
WT
ND1
ATP5a
ATG12
Merge
D6 BVSC(-)
D7 BVSC(-)
D6 BVSC(-)
D7 BVSC(-)
b
D6, 20% O2
ATG12 surface volume/cell (μm3)
10
5
0
***
***
WT
ND1
BVSC(+)
BVSC(-)
c
Conventional protocol
*
****
Modified protocol
n.s.
**

Supplementary Figure 5: Low ATG12 levels in *SC*(-) cells. a, Representative microscopy confocal staining images of ATP5a fluorescence intensity (red) and ATG12 fluorescence intensity (white) of *BV*(+), *BVSC*(+) and *BVSC*(-) cells at D6 and D7 of differentiation of WT and ND1 obtained from conventional (20% oxygen) or modified (from 3% to 20% oxygen) culture conditions. Scale bars = 2 µm. **b**, ATG12 surface volume per cells in *BVSC*(+) and *BVSC*(-) at 20% O_2 of D6 of differentiation. The horizontal lines represent the mean, ***p<0.001, two-way ANOVA with multiple comparisons using Sidak test (WT *BVSC*(+): 11 cells, n=2; WT *BVSC*(-): 13 cells, n=3; ND1 *BVSC*(+): 9 cells, n=2 ND1 *BVSC*(-): 10 cells, n=2). **c**, ATG12 surface volume per cells in *BVSC*(+) and *BVSC*(-) at 20% O_2 at D7 of differentiation (WT *BVSC*(+): 12 cells, n=2; WT *BVSC*(-): 17 cells, n=3; ND1 *BVSC*(+): 11 cells, n=2 ND1 *BVSC*(-): 21 cells, n=3). ATG12 surface volume per cells in *BVSC*(+) and *BVSC*(-) at D7 of differentiation using the modified protocol (WT *BVSC*(+): 12 cells, n=2; WT *BVSC*(-): 14 cells, n=2; ND1 *BVSC*(+): 17 cells, n=3; ND1 *BVSC*(-): 19 cells, n=3). The horizontal line represent the mean, *p<0.05, **p<0.01, ****p<0.0001, two way ANOVA with multiple comparisons using Sidak test.

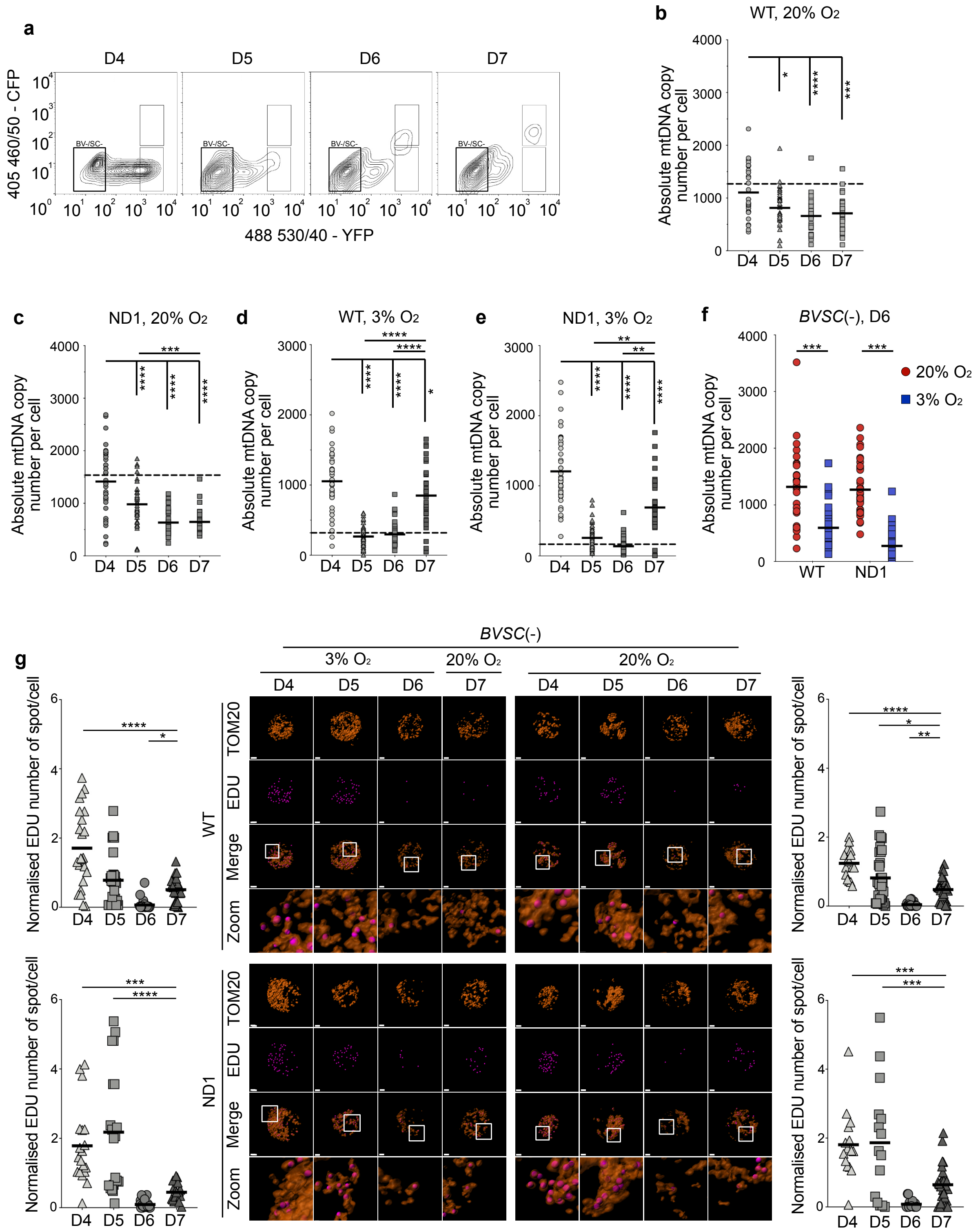

a
D4
D5
D6
D7
405 460/50 - CFP
488 530/40 - YFP
BV-/SC-
b
WT, 20% O2
Absolute mtDNA copy number per cell
c
ND1, 20% O2
d
WT, 3% O2
e
ND1, 3% O2
f
BVSC(-), D6
20% O2
3% O2
WT
ND1
g
BVSC(-)
3% O2
20% O2
TOM20
EDU
Merge
Zoom
WT
ND1
Normalised EDU number of spot/cell

Supplementary Figure 6: A narrower genetic bottleneck in BVSC(-) with reduced mtDNA replication foci number. a, Representative example of contour plots for *BVSC*(-) cells isolation by FACS. Highlighted boxes in the plots show the selected cell population. **b-e**, Number of mtDNA molecules per *BVSC*(-) single cell during WT and ND1 cell differentiation to PGCLC at low and high oxygen concentration. The horizontal lines represent the mean. The dotted lines represent the mean of mtDNA copy number when it is at its minimum in the adjacent *BV* and *BVSC*(+) cells. Data were analysed by one-way ANOVA with multiple comparisons using Tukey test, and p-values are shown as: $p<0.05$, $^{**}p<0.01$, $^{***}p<0.001$ and $^{****}p<0.0001$ (WT cells 3% O_2, D4: 39 cells; D5: 53 cells; D6: 46 cells; D7: 34; ND1 cells 3% O_2, D4: 42 cells; D5: 47 cells; D6: 46 cells; D7: 41 cells; WT cells 20% O_2, D4: 31 cells; D5: 34 cells; D6: 32 cells; D7: 37; ND1 cells 20% O_2, D4: 44 cells; D5: 42 cells; D6: 41 cells; D7: 42 cells from 3 independent differentiations). **f**, mtDNA copy number in WT and ND1 *BVSC*(-) cells at D6 of differentiation at either 20% or 3% O_2. The horizontal lines represent the mean, $^{***}p<0.001$ Two-way ANOVA with multiple comparisons using Sidak test. **g**, Representative microscopy confocal staining images of TOM20 surface (red, upper) and labelled-pulsed with EDU spot (purple, lower) surfaces generated by IMARIS software based on fluorescent intensity signal of *BVSC*(-) cells during PGCLC differentiation. Scale bars = 2 µm. Original images showing the fluorescent intensity are shown in Supplementary Information Fig. S7. Respective quantification of the relative number of EDU spots per cell are shown. Horizontal bars represent the mean (in µm), n=3. $^{*}p<0.05$, $^{**}p<0.01$, $^{***}p<0.001$ and $^{****}p<0.0001$, one-way ANOVA with multiple comparisons using Tukey test (for WT cells 3% O_2, D4: 23 cells, n=3; D5: 28 cells, n=3; D6: 26 cells, n=3; D7: 32, n=3; for ND1 cells 3% O_2, D4: 19 cells, n=3; D5: 20 cells, n=3; D6: 28 cells, n=3; D7: 18, n=3 cells; for WT cells 20% O_2, D4: 16 cells, n=3; D5: 34 cells, n=3; D6: 21 cells, n=3; D7: 28, n=3; for ND1 cells 20% O_2, D4: 15 cells, n=3; D5: 15 cells, n=2; D6: 17 cells, n=3; D7: 29 cells, n=3).

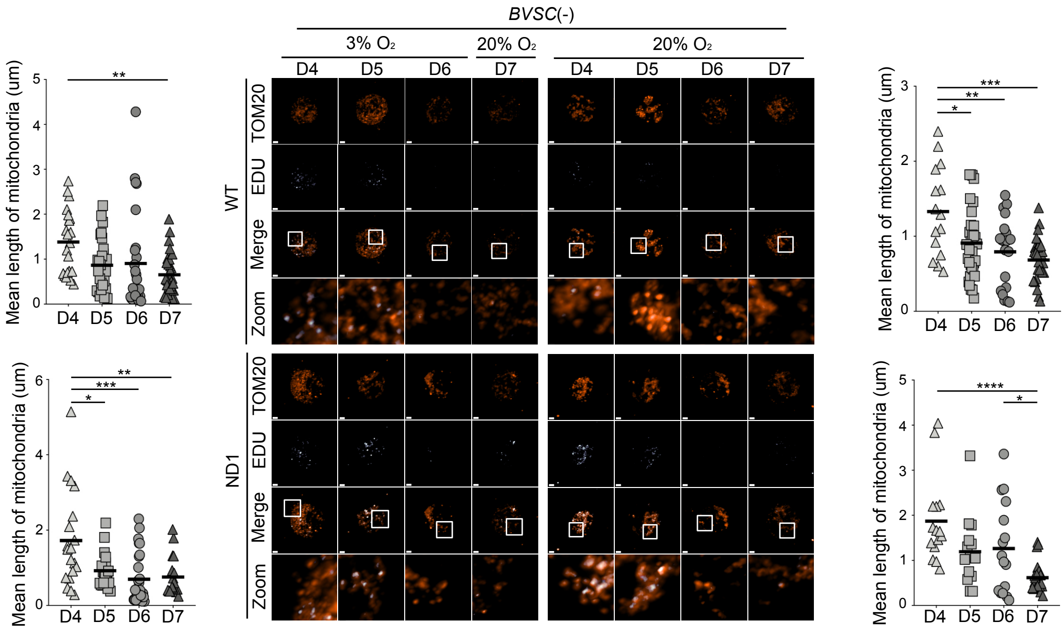

BVSC(-)
3% O2
20% O2
20% O2
D4
D5
D6
D7
WT
ND1
TOM20
EDU
Merge
Zoom
Mean length of mitochondria (um)

Supplementary Figure 7: Mitochondrial fragmentation in BVSC(-) cells harbouring low mtDNA content. Representative microscopy confocal staining images of TOM20 intensity (red, upper) and labelled-pulsed with EDU spot (purple, lower) intensity signal of *BVSC*(-) cells during PGCLC differentiation. Scale bars = 2 μm. Respective quantifications are shown. Horizontal bars represent the mean, *$p<0.05$, **$p<0.01$, ***$p<0.001$ and ****$p<0.0001$, one-way ANOVA with multiple comparisons using Tukey test (WT - 3% O_2: D4: 25 cells, n=3; D5: 24 cells, n=3; D6: 26 cells, n=3; D7: 16 cells, n=2; ND1 - 3% O_2: D4: 19 cells, n=3; D5: 22 cells, n=3; D6: 24 cells, n=3; D7: 13 cells, n=2; (WT - 20% O_2: D4: 20 cells, n=3; D5: 25 cells, n=3; D6: 15 cells, n=3; D7: 27 cells, n=3; ND1 - 20% O_2: D4: 23 cells, n=3; D5: 10 cells, n=2; D6: 9 cells, n=2; D7: 20 cells, n=3).

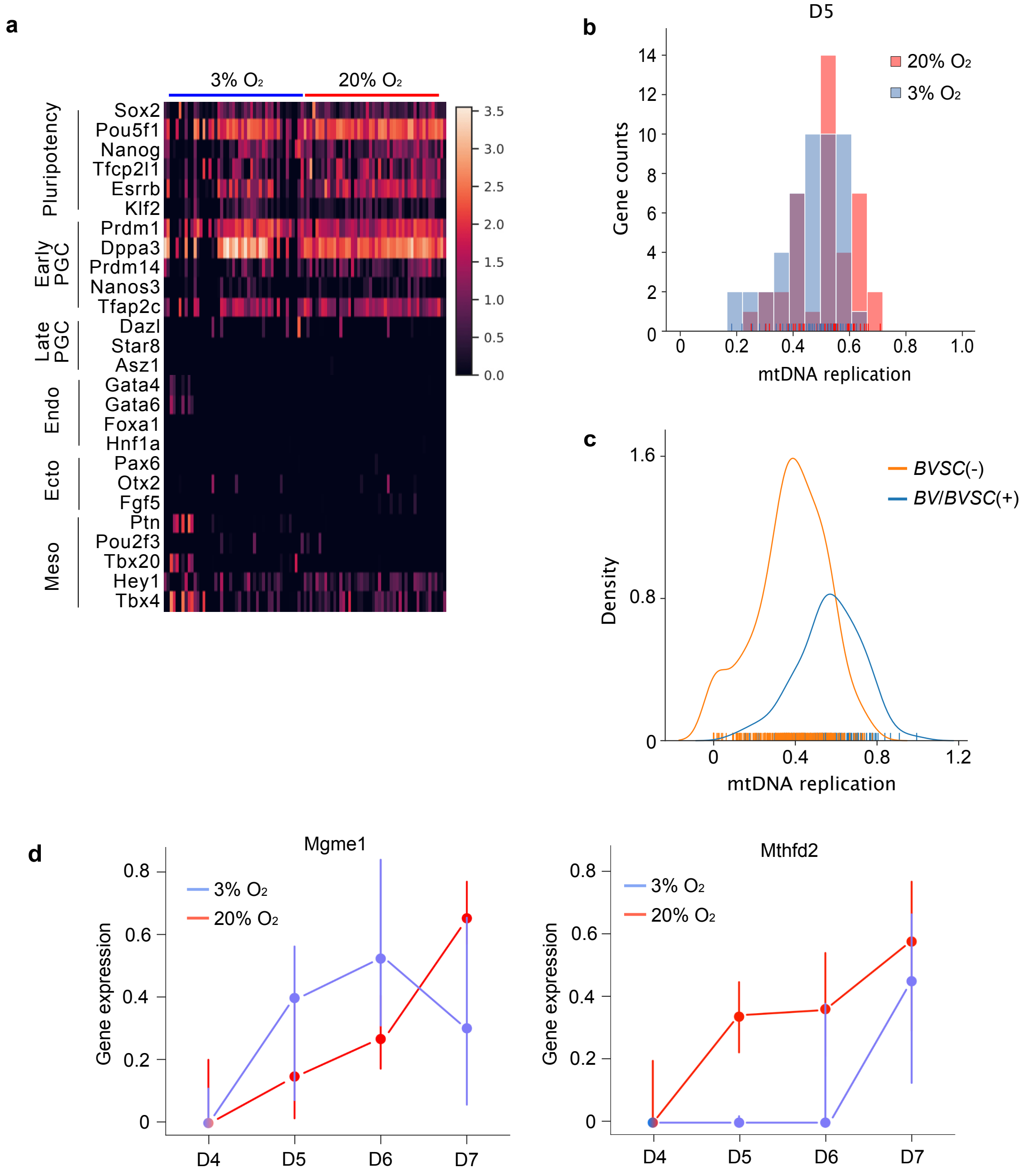
a
3% O2
20% O2
Pluripotency
Early PGC
Late PGC
Endo
Ecto
Meso
Sox2
Pou5f1
Nanog
Tfcp2l1
Esrrb
Klf2
Prdm1
Dppa3
Prdm14
Nanos3
Tfap2c
Dazl
Star8
Asz1
Gata4
Gata6
Foxa1
Hnf1a
Pax6
Otx2
Fgf5
Ptn
Pou2f3
Tbx20
Hey1
Tbx4
3.5
3.0
2.5
2.0
1.5
1.0
0.5
0.0
b
D5
20% O2
3% O2
Gene counts
mtDNA replication
c
BVSC(-)
BV/BVSC(+)
Density
mtDNA replication
d
Mgme1
Mthfd2
3% O2
20% O2
Gene expression
D4
D5
D6
D7

Supplementary Figure 8: Single cell RNA sequencing of *BVSC*(+/-) cells during *in vitro* differentiation. **a**, Heatmap showing the gene expression of *BVSC*(+) at D7, after differentiation using the conventional (20% O_2: 48 cells) or modified protocol (3%O_2: 48 cells). Endo=Endoderm, Ecto=Ectoderm, Meso=Mesoderm. **b**, Bar plot showing the number of mtDNA replication related genes expressed per cell (mtDNA replication score, x axis) during PGCLC differentiation using the conventional protocol (red, 85 cells) and modified protocol (blue, 96 cells). The mtDNA replication score is calculated based on the expression of 18 genes (Supplementary Table 2), p-value=0.01 with Wilcoxon rank-sum tests were applied with significance threshold of 0.05 for DEG discovery and Benjamini–Hochberg procedure were used to obtain multiple-testing corrected p-values. **c**, Line plot showing the number of mtDNA replication related genes expressed per *BVSC*(-) cell (mtDNA replication score, x axis) during PGCLC differentiation in comparison to *BVSC*(+) cells from D5 to D7. The mtDNA replication score is calculated based on the expression of 18 genes (Supplementary Table 2), p-value=$7.5x10^{-22}$ with Wilcoxon rank-sum tests were applied with significance threshold of 0.05 for DEG discovery and Benjamini–Hochberg procedure were used to obtain multiple-testing corrected p-values. **d**, *Mgme1* and *Mthfd2* gene expression during PGCLC differentiation at 20% and 3% oxygen concentration. Data represent the median of read counts ± SD, Wilcoxon rank-sum tests were applied with significance threshold of 0.05 for DEG discovery and Benjamini–Hochberg procedure were used to obtain multiple-testing corrected p-values.

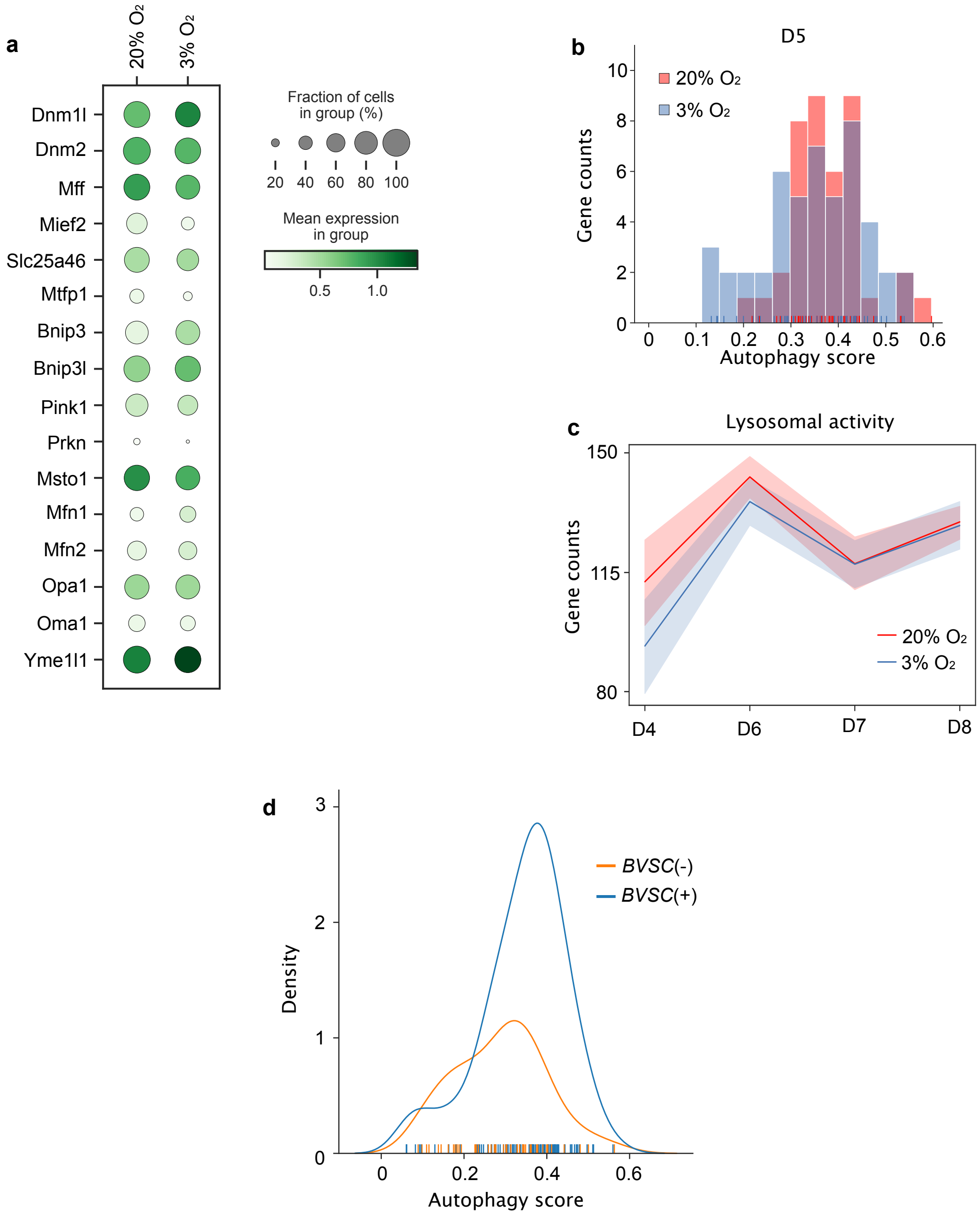
a
20% O2
3% O2
Dnm1l
Dnm2
Mff
Mief2
Slc25a46
Mtfp1
Bnip3
Bnip3l
Pink1
Prkn
Msto1
Mfn1
Mfn2
Opa1
Oma1
Yme1l1
Fraction of cells in group (%)
20 40 60 80 100
Mean expression in group
0.5 1.0
b
D5
20% O2
3% O2
Gene counts
Autophagy score
c
Lysosomal activity
Gene counts
20% O2
3% O2
D4 D6 D7 D8
d
BVSC(-)
BVSC(+)
Density
Autophagy score

Supplementary Figure 9: Single cell RNA sequencing in *BVSC(+/-)* cells shows no sign of autophagy related gene expression during the in vitro genetic bottleneck. a, Dot plots comparing the expression of genes in PGCLC at D5 involved in mitochondrial dynamics in cells differentiated in either a low or high oxygen concentration. **b**, Bar plot showing the number of autophagy related genes expressed per cell (autophagy score, x axis) during PGCLC differentiation using the conventional protocol (red, 85 cells) and modified protocol (blue, 96 cells). The autophagy score is calculated based on the expression of 36 genes (Supplementary Table 2) with Wilcoxon rank-sum tests were applied with significance threshold of 0.05 for DEG discovery and Benjamini–Hochberg procedure were used to obtain multiple-testing corrected p-values. **c**, Line plot with error bands showing the expression of genes regulating lysosomal function and biosynthesis, a total of 464 genes considered (Supplementary Table 2) during PGCLC differentiation. **d**, Line plot showing the number of autophagy related genes expressed per cell (autophagy score, x axis) during PGCLC differentiation comparing *BVSC*(-) to *BVSC*(+) cells at D7. The autophagy score is calculated based on the expression of 18 genes (Supplementary Table 2), p-value=0.0005 with Wilcoxon rank-sum tests were applied with significance threshold of 0.05 for DEG discovery and Benjamini–Hochberg procedure were used to obtain multiple-testing corrected p-values.
