# Supplementary material for: Oxygen tension modulates the mitochondrial genetic bottleneck and influences the segregation of a heteroplasmic mtDNA variant in vitro
Source: Commun Biol. 2021 May 14;4:584. doi: 10.1038/s42003-021-02069-2 (PMC8121860; doi:10.1038/s42003-021-02069-2)
Supplement: Supplementary file 2 — Description of Additional Supplementary Files. [file 42003_2021_2069_MOESM2_ESM.pdf]

Description of Additional Supplementary Files

Supplementary Video Legend

Supplementary Video 1

3D reconstitution of a representative microscopy confocal staining image of TOM20 intensity (red) and labelled-pulsed with EDU intensity (white) acquired using a piezo Z-stage (0.2 µm stack) at D7 of WT PGCLC differentiation.

Supplementary Data Legends

Supplementary Data 1

Raw data for mtDNA copy number, heteroplasmy level of the mt-ND1 variant, number of replicating foci, ATG12 level and mitochondria 3D distribution score per cell.

Supplementary Data 2

List of genes used to calculate the score reflecting the number of mtDNA replication, autophagy, lysosome related genes expressed per cell.
